# Fragile X mental retardation 1 gene FMR1 promotes proliferation, migration, and invasion of gastric cancer cells via c-MYC

**DOI:** 10.1186/s12967-025-07140-8

**Published:** 2025-11-03

**Authors:** Yiqian Han, Chenxi Mao, Kangjie Zhou, Mingtong Liang, Luming Zhao, Yidong Hong, Jingzhou Zhang, Nan Hu, Fenglei Wu

**Affiliations:** 1 Lianyungang Clinical College of Nanjing Medical University, Lianyungang, 222000 China; 2 The Affiliated Lianyungang Hospital of Xuzhou Medical University, Lianyungang, 222000 China

**Keywords:** FMR1, c-MYC, Gastric cancer, Single-cell analyses, Proliferation, Migration, Invasion

## Abstract

**Background:**

Gastric cancer is a highly aggressive malignancy with poor prognosis and low survival rates. The Fragile X Mental Retardation 1 (FMR1) gene has been implicated in the development and progression of various tumors, but its role in gastric cancer remains unclear.

**Methods:**

We performed pan-cancer expression analysis of FMR1 using the TIMER2.0 platform, and evaluated its differential expression in gastric cancer versus normal gastric tissues in the TCGA cohort. FMR1 expression was validated by qRT-PCR, Western blotting, and immunohistochemistry. Using R software and clinical samples to evaluate the association between FMR1 expression levels and clinicopathological factors in gastric cancer patients, and to analyze patient survival curves. The relationship between FMR1 expression and tumor immune infiltration was analyzed via the TISIDB database, and after co-culture, cytokine secretion by CD4⁺ T cells was assessed using ELISA following FMR1 knockdown in tumor cells. Functional enrichment analyses of FMR1 and its interacting genes were performed. Single-cell transcriptomics was used to extend the interpretation of intratumoral lineages and states. Malignant epithelial populations were identified using inferCNV, and these cells were subsequently stratified by FMR1 expression for GSVA. We measured FMR1 expression in control and FMR1 knockdown gastric cancer cells, performed proliferation, migration, and invasion assays to investigate the biological effects of FMR1 in gastric cancer. Mechanistic insights were further explored through co-immunoprecipitation, cycloheximide chase, proteasome inhibition, and rescue assays.

**Results:**

FMR1 was significantly overexpressed in gastric cancer tissues compared to normal gastric mucosa, with high expression levels associated with poor prognosis. The differential expression of FMR1 in gastric cancer was strongly associated with the activity of multiple immune cell types within the tumor microenvironment. Functional assays further demonstrated that FMR1 knockdown suppressed cytokine secretion by CD4⁺ T cells. The expression level of FMR1 in malignant epithelial cells is higher than that in the non—malignant group, and the high—expression group of FMR1 in malignant cells shows a consistent increase in the Hallmark gene sets directly related to stem cell like phenotype, chemotherapy resistance, and immune evasion. Knockdown of FMR1 suppressed gastric cancer cell proliferation, migration, and invasion, while mechanistic studies indicated that FMR1 positively regulates c-MYC expression to drive these phenotypes. And we found that FMR1 interacted with c-MYC at the protein level and stabilized c-MYC by suppressing its proteasomal degradation.

**Conclusion:**

Our findings demonstrate that FMR1 promotes gastric cancer cell proliferation, migration, and invasion through c-MYC signaling, suggesting that FMR1 may serve as a potential prognostic biomarker and therapeutic target for gastric cancer.

**Supplementary Information:**

The online version contains supplementary material available at 10.1186/s12967-025-07140-8.

## Introduction

Gastric cancer (GC) is one of the most common malignant tumors worldwide, with over one million new cases annually [1], accounting for 5.6% of all cancer diagnoses [2]. Despite advances in treatment, GC remains highly lethal due to its insidious onset and lack of specific early symptoms, which often results in late-stage diagnosis and limited therapeutic options. Although early-stage GC can be managed surgically, most patients are diagnosed at advanced stages when curative resection is not feasible, complicating treatment and leading to poor prognosis.

Recent research has improved our understanding of GC pathogenesis, but identifying aberrantly expressed molecular markers and actionable therapeutic targets remains essential for early detection and precision therapy. Targeted therapies have become standard first-line options for cancers harboring driver mutations, and multi-targeted strategies have demonstrated superior efficacy compared to single-agent treatments [3]. Therefore, elucidating the molecular mechanisms underlying GC progression is crucial for discovering novel therapeutic targets and optimizing combination treatment strategies.

The Fragile X Mental Retardation 1 (FMR1) gene, a member of the Fragile X Related (FXR) gene family, was first identified in 1991. FMR1 is a highly conserved gene located on the long arm of the X chromosome (Xq27.3), spanning approximately 40 kb and comprising 17 exons. It produces an around 4 kb mRNA that encodes the fragile X mental retardation protein (FMRP), which is 632 amino acids in length with a molecular weight of approximately 69 kDa [4]. While extensively studied in neuroscience due to its established role in fragile X syndrome—the most common inherited form of intellectual disability and syndromic autism—FMRP is ubiquitously expressed and influences diverse biological processes in multiple tissues [4].

For example, Yuhan Hu et al. [5] demonstrated that FMR1 promotes colorectal cancer progression by stabilizing EGFR mRNA in an m6A-dependent manner. FMRP facilitates hepatocellular carcinoma metastasis by regulating the localization and local translation of STAT3 mRNA at cellular protrusions [6]. E. Caredda et al. [7] further reported that FMRP levels may serve as a prognostic factor for site-specific metastasis in primary breast cancer. In addition, other studies have shown that FMRP plays a critical role in glioblastoma progression through modulation of the WNT signaling pathway [8]. In-depth investigation of the regulatory mechanisms of FMR1 in tumor biology is crucial for the clinical implementation of targeted therapeutic strategies against FMR1.However, the expression and functional significance of FMR1 in gastric cancer remain largely unknown.

In this study, we analyzed FMR1 expression in gastric cancer tissues and normal gastric mucosal tissue, examined its relationship with clinicopathological features and patient prognosis, and explored its role in tumor immune microenvironment modulation. Single-cell analyses provided insights into FMR1 expression patterns in malignant epithelial cells. We further analyzed that high FMR1 expression is associated with stem cell like phenotype, chemotherapy resistance, and immune evasion. We further investigated the biological effects of FMR1 on gastric cancer cell proliferation, migration, and invasion in vitro, and elucidated the underlying mechanism involving c-MYC signaling. These findings may support FMR1 as a novel biomarker and therapeutic target in gastric cancer.

## Materials and methods

### Differentially expressed, survival, and functional analysis

We analyzed FMR1 expression across multiple cancer types using TIMER2.0. Differential expression between gastric cancer and normal gastric tissues was evaluated using data from The Cancer Genome Atlas (TCGA, https://portal.gdc.cancer.gov/). The selected samples are from the RNA-seq data in the 3-level HTSeq-FPKM (Fragments Per Kilobase per Million) format and clinical data (including age, gender, grade, TNM stage, etc.) of the TCGA-STAD project. Survival analysis was conducted via the Kaplan–Meier Plotter database. Associations between FMR1 expression and clinicopathological features in gastric cancer patients were assessed using R software. The relationship between FMR1 expression and immune infiltration in gastric cancer was analyzed via TISIDB. Functional enrichment of FMR1 and its interacting genes was performed using established annotation tools.

### Single-cell analysis

Single-cell RNA sequencing data from the gastric cancer GEO dataset (GSE183904) included 26 tumor samples and 10 normal samples. Data preprocessing was conducted using Seurat (v5.0.1), excluding low-quality cells with > 10% mitochondrial gene expression or < 200 or > 5000 detected genes. After normalization, 2000 highly variable genes were selected for principal component analysis (PCA), and the top 20 principal components were retained. Then, batch effect correction was performed using Harmony. Subsequently, cell clustering and visualization were carried out. Marker genes include: fibroblasts (COL1A2, DCN), epithelial cells (EPCAM, KRT19), T cells (CD3D, CD3E), B cells (CD79A, MS4A1), endothelial cells (PECAM1, VWF), myeloid cells (CD163, MRC1), plasma cells (IGHA1, IGHG1), mast cells (KIT, TPSB2), and proliferating cells (TOP2A, CENPF). Differentially expressed genes (DEGs) were selected using |log_2_FC|> 0.25 and adjusted *p* value < 0.05. Perform GO biological process enrichment analysis on the DEGs, and present the results through a bubble chart. Using normal epithelial cells as a reference, CNVs were inferred for the remaining cells with inferCNV (v1.20.0; cutoff = 0.1; cluster_by_groups = TRUE; denoise = TRUE; HMM = TRUE; output = infercnv_result). A normalized expression matrix (neutral baseline = 1) was obtained, and a CNV score for each cell was calculated as CNVscore = mean((CNV–1)^2) [9–11]. The 90th percentile (P90) of CNV scores in the reference group was used as the threshold [12], with cells having CNVscore ≥ P90 defined as candidate malignant epithelial cells and the remainder classified as non-malignant epithelial cells.

### Gene set variation analysis (GSVA)

Malignant epithelial cells were identified in Seurat, and the log-normalized expression matrix was extracted. The pathway activities of the high- and low-expression FMR1 groups in malignant cells were obtained by using GSVA combined with MSigDB Hallmark. Here, we focused on the gene sets related to stem cell like phenotype, chemotherapy resistance, and immune evasion in the pathways. Based on the literature, our gene set screening was as follows: stem cell like phenotype (HALLMARK_MYC_TARGETS_V1 [13], HALLMARK_WNT_BETA_CATENIN_SIGNALING [14]), chemotherapy resistance (HALLMARK_DNA_REPAIR [15], HALLMARK_UNFOLDED_PROTEIN_RESPONSE [16], HALLMARK_PI3K_AKT_MTOR_SIGNALING [17], HALLMARK_MTORC1_SIGNALING [18], HALLMARK_TNFA_SIGNALING_VIA_NFKB [19]), and immune evasion (HALLMARK_TGF_BETA_SIGNALING [20], HALLMARK_WNT_BETA_CATENIN_SIGNALING [14]). Pathway activity was evaluated, enrichment differences between FMR1^high and FMR1^low groups were compared, and results were visualized with bar and violin plots. Statistical significance was assessed using Wilcoxon or t tests with FDR correction.

### Cell culture

Some human gastric cancer cell lines (AGS, HGC-27) and the normal gastric epithelial cell line (GES-1) were obtained from Shanghai Institute of Biochemistry and Cell Biology(Shanghai, China), and the other human gastric cancer cell lines (MKN-74, SNU-484) were obtained from Fu Heng Cell Center (Shanghai, China). Cells were cultured in RPMI 1640(KeyGEN, Nanjing, China) medium supplemented with 10% fetal bovine serum (FBS, Clark, Shanghai, China), 100 U/mL penicillin, and 100 U/mL streptomycin at 37 °C in 5% CO_2_. Cells were passaged at 80–90% confluence using trypsin(VICMED, Xuzhou, China) digestion.

### Co-culture

Peripheral blood mononuclear cells (PBMCs) were isolated from healthy donor peripheral blood by Ficoll density gradient centrifugation. CD4⁺ T cells were obtained at high purity through negative selection using a magnetic cell sorting system (Real-gen Biotechnology, Suzhou, China) and subsequently activated. Gastric cancer cells and CD4⁺ T cells were directly co-cultured at a 1:10 (tumor:T cell) ratio for 24 or 48 h. Culture supernatants were then collected, and the levels of TNF-α, IFN-γ, and IL-4 were measured using ELISA kits (Proteintech, Wuhan, China).

### Small interfering RNA (siRNA) transfection

Small interfering RNAs (siRNAs) targeting FMR1 and c-MYC, along with control scrambled siRNA, were synthesized by GenePharma (Wuhan, China). Transfections were performed using Lipofectamine 2000 (Invitrogen, Thermo Fisher Scientific, MA, USA) according to the manufacturer’s instructions. The oligonucleotides used are as follows:

- si FMR1 #1, 5′- AAAGCTATGUGACUGAUGA-3′;
- si FMR1 #2, 5′- CAGCUUGCCUCGAGAUUUC-3′;
- si FMR1 #3, 5′- GACAUGCACUUUCGGAGUC-3′.
- si c-MYC #1, 5′- GAGGAGACAUGGUGAACCA -3′;
- si c-MYC #2, 5′- GGGUCAAGUUGGACAGUGU -3′.

### Lentivirus infection

To achieve stable overexpression or knockdown of FMR1 and c-MYC in SNU-484 cells, the full-length coding sequences of human FMR1 and c-MYC, or specific shRNA sequences targeting human FMR1, were cloned into the lentiviral vector pCDH-CMV-MCS-EF1-copGFP (Sangon Biotech, Shanghai, China).HEK293T cells(Shanghai Institute of Biochemistry and Cell Biology, Shanghai, China) were co-transfected with expression and packaging plasmids (psPAX2, pMD2.G) at a 4:3:1 ratio using Lipofectamine 2000. Viral supernatants were collected and used to infect SNU-484 cells in the presence of 8 μg/mL polybrene. pCDH-CMV-FMR1, pCDH-CMV-c-MYC and specific shRNA sequences targeting human FMR1 were purchased from Sangon Biotech (Shanghai, China).

### Tissue samples and patients

Seventy-eight pairs of gastric cancer and matched adjacent normal tissues archived in 2020 were obtained from Lianyungang Clinical College of Nanjing Medical University. Inclusion criteria: patients had not received chemotherapy, radiotherapy, or other antitumor treatments prior to surgery; postoperative pathological diagnosis confirmed gastric cancer by the Department of Pathology of our hospital; paraffin-embedded tissue specimens were available; patients had undergone radical gastrectomy; and informed consent was obtained. Exclusion criteria: patients with cachectic diseases involving major organs such as the liver or kidney; patients with other primary malignant tumors of major organs; and patients who declined to participate in the study.All procedures adhered to the Declaration of Helsinki, with informed consent obtained from all patients. The study was approved by the institutional ethics committee (approval no. KY-20250709001.A01).

### RNA isolation, reverse transcription, and qRT-PCR

Total RNA was extracted using an RNA Purification Kit (NCM Biotech, Suzhou, China). Reverse transcription was performed with RT Master Mix for qPCR(NCM Biotech, Suzhou, China). qRT-PCR was conducted using SYBR Green Master Mix (NCM Biotech, Suzhou, China) on an ABI 7500 system. Relative mRNA levels were calculated using the 2^–ΔΔCT method with GAPDH as the internal control. The primers used in this study are as follows: FMR1 (F: GGTCAAGGAATGGGTCGAGG, R: AGTTCGTCTCTGTGGTCAGAT);c-Myc(F: GCGTCCTGGGAAGGGAGATC.

CGGAGC,R:TTGAGGGGCATCGTCGCGGGAGGCTG);GAPDH(F:AGCCACATCGCTCAGACAC, R: GCCCAATACGACCAAATCC).

#### Immunohistochemistry

Paraffin-embedded tissue sections underwent deparaffinization, hydration, antigen retrieval, and incubation with anti-FMR1 antibody (ab17722, 1:1000, Abcam, Cambridge, MA, USA). The data were interpreted by two senior pathologists. Interpretation of staining results: Positive FMR1 staining was mainly concentrated in the cytoplasm. Staining intensity and percentage of positive cells were scored. Score according to the percentage of positive cells: score 0 for ≤ 5%, score 1 for 6–25%, score 2 for 26–50%, score 3 for 51–75%, and score 4 for over 75%. Score according to the staining intensity of positive cells: score 0 for unstained, score 1 for light yellow, score 2 for brownish—yellow, and score 3 for brownish—tan. The final immunoreactivity score was calculated as the product of these scores: ≤ 2 is defined as negative expression, and > 2 is defined as positive expression.

#### Western blot analysis

Cells were lysed in RIPA buffer(Proteintech, Wuhan, China) containing protease inhibitors(VICMED, Xuzhou, China). The BCA Protein Assay Kit (NCM Biotech, Suzhou, China) is used for protein quantification. Proteins were separated by SDS-PAGE, transferred to PVDF membranes(Millipore, MA, USA). Incubate each PVDF membrane in a blocking buffer (NCM Biotech, Suzhou, China) at room temperature for 10 min, and then incubate it with a specific primary antibody overnight at 4°C. The next day, incubate the PVDF membrane with an HRP-conjugated Affinipure goat anti-rabbit IgG (H + L) antibody (SA00001-2, 1:5000; Proteintech, Wuhan, China) or Affinipure goat anti-mouse IgG (H + L) antibody (SA00001-1, 1:5000; Proteintech, Wuhan, China) at room temperature for 1 h. Visualize the protein bands using an ECL kit (Proteintech, Wuhan, China) and detect them using a bioimaging system (Proteinsimple, SV, USA). Calculate the relative protein levels after normalizing to the levels of GAPDH, which is used as a loading control. Analyze and organize the results using Image J software. The primary antibodies used include anti-FMR1 (ab 17,722, 1:1000, Abcam, Cambridge, MA, USA), anti-c-MYC (10,828—1—AP, 1:1000, Proteintech, Wuhan, China), anti-FLAG (66,008–4-Ig, 1: 20,000, Proteintech,Wuhan, China) and anti-GAPDH (10,494-1-AP, 1:5000, Proteintech, Wuhan, China).

#### Co-immunoprecipitation

oeGFP and oeFMR1 cells were lysed on ice for 30 min in IP lysis buffer (Thermo Fisher Scientific, Shanghai, China). To investigate the interaction between FMRP and c-MYC, the lysates were incubated overnight at 4 °C with magnetic beads conjugated to anti-Flag antibody (Selleckchem, TX, USA). Beads were collected using a magnetic rack, washed three times with wash buffer, and resuspended in loading buffer. Samples were then heated at 95 °C for 5 min, and the supernatants were subjected to Western blot analysis.

#### Cycloheximide chase experiment

SNU-484 cells transfected with shNC or shFMR1 were treated with cycloheximide (CHX, 100 μg/mL; MCE, NJ, USA) at different time points. Cells were harvested at indicated intervals (0, 0.25, 0.5, and 1 h), and total protein was extracted. Western blotting was then performed to assess the time-dependent changes in c-MYC protein levels in the two groups, thereby evaluating the effect of FMR1 on c-MYC protein stability.

#### Proteasome inhibition experiment

SNU-484 cells transfected with shNC or shFMR1 were treated with the proteasome inhibitor MG132 (10 μM; MCE, NJ, USA) for 6 h prior to harvest. Total protein was extracted, and c-MYC expression levels were examined by Western blotting in the two groups.

#### Cell proliferation assays

CCK-8 assays were performed by seeding transfected cells into 96-well plates, and culture in a medium containing 10% FBS. After 24, 48, 72, and 96 h, add CCK—8 reagent (NCM Biotech, Suzhou, China) (10 μl/well) and incubate for 1 h. Measure the optical density at 490 nm to generate a growth curve.

For the colony formation assay, the transfected cells were seeded into 6-well plates (1000 cells/well). The medium was changed every 3 days. After 10—15 days of culture, the cells were washed with phosphate-buffered saline, fixed with 4% paraformaldehyde for 20 min, stained with crystal violet for 20 min, and the number of colonies with more than 50 cells was counted. All experiments were performed in triplicate.

#### Wound healing assays

Cells were grown to confluence in 6-well plates. Scratch wounds were created with pipette tips. Observe the wound healing within the scratch line at the specified time point and take photos of the representative scratch lines. Use Photoshop software to optically measure the wound size. All experiments were performed in triplicate.

#### Transwell cell invasion assays

Transwell chambers (Corning, NY,USA) with 8 μm pores were coated with 60 μl Matrigel(1:8 dilution, BD Bioscience). Transfected cells were seeded in serum-free medium in the upper chamber; medium with 20% FBS was placed in the lower chamber. After incubation, invaded cells were fixed, stained, and counted microscopically.

#### Statistical analysis

Data were analyzed using GraphPad Prism 10 and SPSS 27. Comparisons were made using unpaired two-tailed t-tests. Pearson chi-square test was used to analyze the correlation of clinicopathological parameters. Significance markers are: ns, *P* ≥ 0.05; * *P* < 0.05; ** *P* < 0.01; *** *P* < 0.001; **** *P* < 0.0001.

## Results

### Pan-cancer analysis of FMR1 expression

**Fig. 1:**
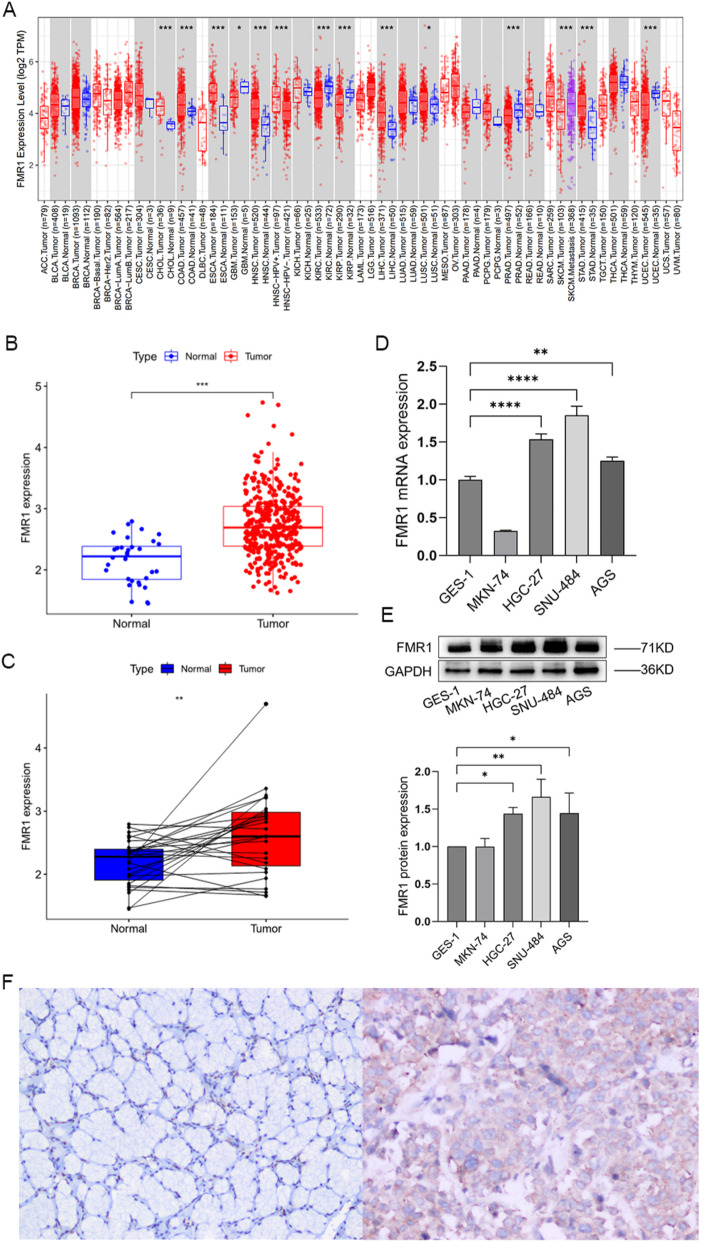
Expression levels of FMR1. **A** Differential expression of FMR1 across pan-cancer. **B** Differential expression of FMR1 in unpaired gastric cancer samples from the TCGA database. **C** Differential expression of FMR1 in paired gastric cancer samples from the TCGA database. **D** FMR1 mRNA expression levels in normal versus gastric cancer cell lines.** E** FMR1 protein expression levels in normal versus gastric cancer cell lines. **F** Immunohistochemical staining of FMRP expression in normal tissues adjacent to gastric cancer and gastric cancer tissues (SP, × 200)

Pan-cancer analysis revealed that FMR1 mRNA expression was significantly upregulated in several cancer types, including cholangiocarcinoma (CHOL, *P* < 0.001), colon adenocarcinoma (COAD, *P* < 0.001), esophageal carcinoma (ESCA, *P* < 0.001), head and neck squamous cell carcinoma (HNSC, *P* < 0.001), lung squamous cell carcinoma (LUSC, *P* < 0.05), liver hepatocellular carcinoma (LIHC, *P* < 0.001), and stomach adenocarcinoma (STAD, *P* < 0.001) (Fig. 1A). Conversely, FMR1 was downregulated in glioblastoma multiforme (GBM, *P* < 0.05), kidney clear cell carcinoma (KIRC, *P* < 0.001), kidney papillary cell carcinoma (KIRP, *P* < 0.001), prostate adenocarcinoma (PRAD, *P* < 0.001), and uterine corpus endometrial carcinoma (UCEC, *P* < 0.001). No significant differences were observed between tumor and normal tissues in bladder cancer (BLCA), breast cancer (BRCA), cervical squamous cell carcinoma (CESC), kidney chromophobe (KICH), lung adenocarcinoma (LUAD), pancreatic adenocarcinoma (PAAD), pheochromocytoma and paraganglioma (PCPG), rectal adenocarcinoma (READ), and thyroid carcinoma (THCA), suggesting tumor-specific regulatory mechanisms. Additionally, FMR1 mRNA expression was positively correlated with HPV-associated HNSC and metastatic skin cutaneous melanoma (SKCM) (*P* < 0.001) (Fig. 1A), indicating that FMR1 may play an important role in the tumorigenesis and progression of diverse cancers.

### Overexpression of FMR1 in gastric cancer cell lines and tissues

Analysis of TCGA data demonstrated significantly higher FMR1 mRNA expression in gastric cancer tissues compared to normal gastric mucosa in both unpaired (*P* < 0.001; Fig. 1B) and paired samples (*P* < 0.01; Fig. 1C). Validation experiments using qRT-PCR and Western blotting showed significantly elevated FMR1 expression in human gastric cancer cell lines (MKN-74, HGC-27, SNU-484, AGS) compared with the normal gastric epithelial cell line GES-1 (*P* < 0.001), with the highest expression in SNU-484 cells (Fig. 1D,E). Immunohistochemistry was used to detect the expression of FMR1 in 78 pairs of gastric cancer tissues and their corresponding adjacent normal tissues in our hospital. The results showed that FMR1 was mainly expressed in the cytoplasm, and the protein expression level of FMR1 in gastric cancer was higher than that in adjacent normal tissues, and the difference was statistically significant (χ^2^ = 6.915, *P* = 0.009; Fig. 1F, Table 1). These findings suggest that increased FMR1 expression may be involved in gastric tumorigenesis.

**Table 1:** Comparison of the positive rate of FMRP expression in gastric cancer tissues and adjacent tissues

Type	FMRP
Gastric cancer tissue	72 (92%)
Peritumoral tissue	67 (86%)
$\chi^{2}$	6.915
*P*	0.009

### Association between FMR1 expression and clinicopathological features and prognosis in gastric cancer

**Fig. 2:**
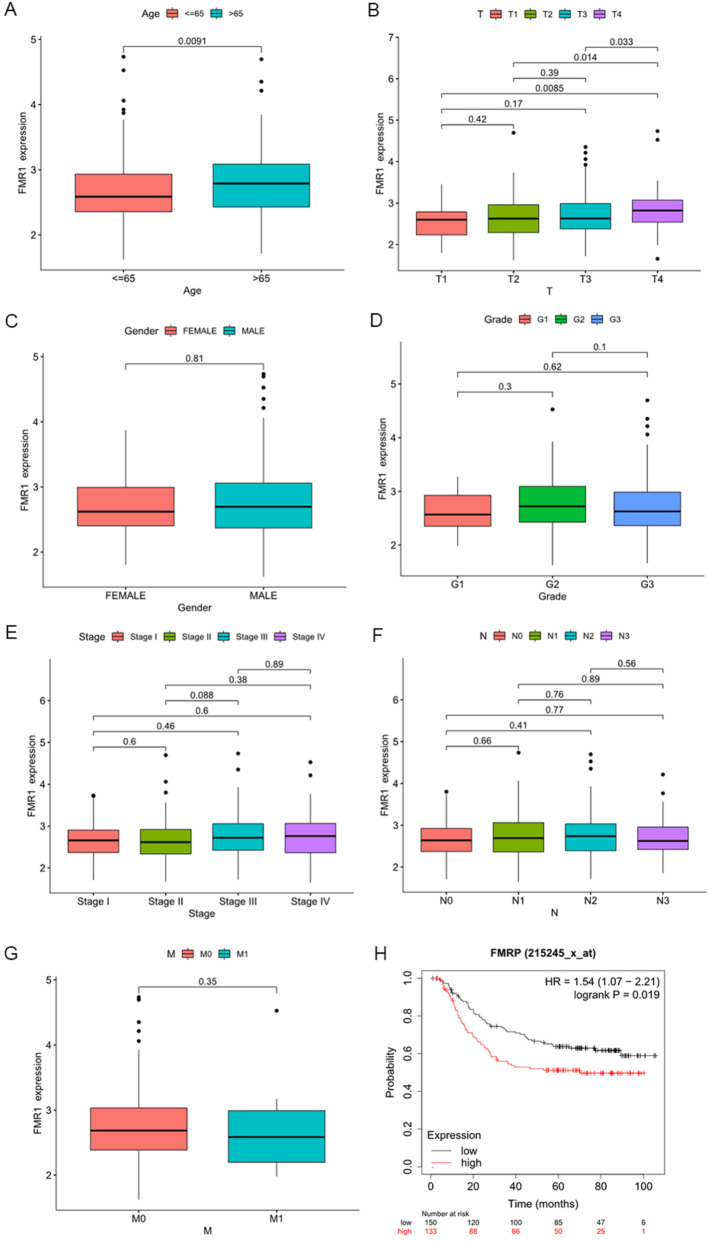
Association between FMR1 expression and clinicopathological features and prognosis in gastric cancer. **A** Correlation between FMR1 expression and patient age. **B** Correlation between FMR1 expression and T stage. **C** Correlation between FMR1 expression and sex. **D** Correlation between FMR1 expression and histological grade. **E** Correlation between FMR1 expression and clinical stage. **F** Correlation between FMR1 expression and N stage. **G** Correlation between FMR1 expression and M stage. **H** Relationship between FMR1 expression levels and overall survival in gastric cancer patients

We next investigated the relationship between FMR1 expression and clinicopathological parameters in gastric cancer. Bioinformatic analyses revealed significant positive correlations between FMR1 expression and both patient age and T stage (*P* < 0.05; Fig. 2A,B), with no significant associations with other clinical features (Fig. 2C–G). Immunohistochemistry further indicated that high FMR1 expression was significantly associated with lymph node metastasis and advanced TNM stage (*P* < 0.05), but not with age, sex, differentiation, invasion depth, or tumor diameter (Table 2). Survival analysis using the online Kaplan–Meier Plotter database revealed that patients with high FMR1 expression had significantly worse overall survival compared with those with low expression (hazard ratio [HR] = 1.54, 95% confidence interval [CI]: 1.07–2.21, *P* = 0.019; Fig. 2H). These results suggest that FMR1 overexpression is closely associated with poor prognosis in gastric cancer and may serve as a prognostic biomarker.

**Table 2:** Relationship between FMRP expression and clinicopathological features

Clinical pathological	Number	FMRP	
		+	*P*
Age (year)			0.161
≥ 65	47	45	
< 65	31	27	
Gender			0.808
Male	62	57	
Female	16	15	
Degree of differentiatio			
Low	32	28	
Medium	42	40	
High	4	4	
Degree of influtration			0.212
Mucosa layer	2	2	
Muscle layer	17	14	
Serous layer	59	56	
Lymph node metastasis			0.012
Yes	59	57	
No	19	15	
TNM staging			0.008
I	8	5	
II	27	25	
III	35	34	
IV	8	8	
Tumor size (cm)			0.401
≥ 5	25	24	
< 5	53	48	

### Functional enrichment analysis of FMR1 in gastric cancer

**Fig. 3:**
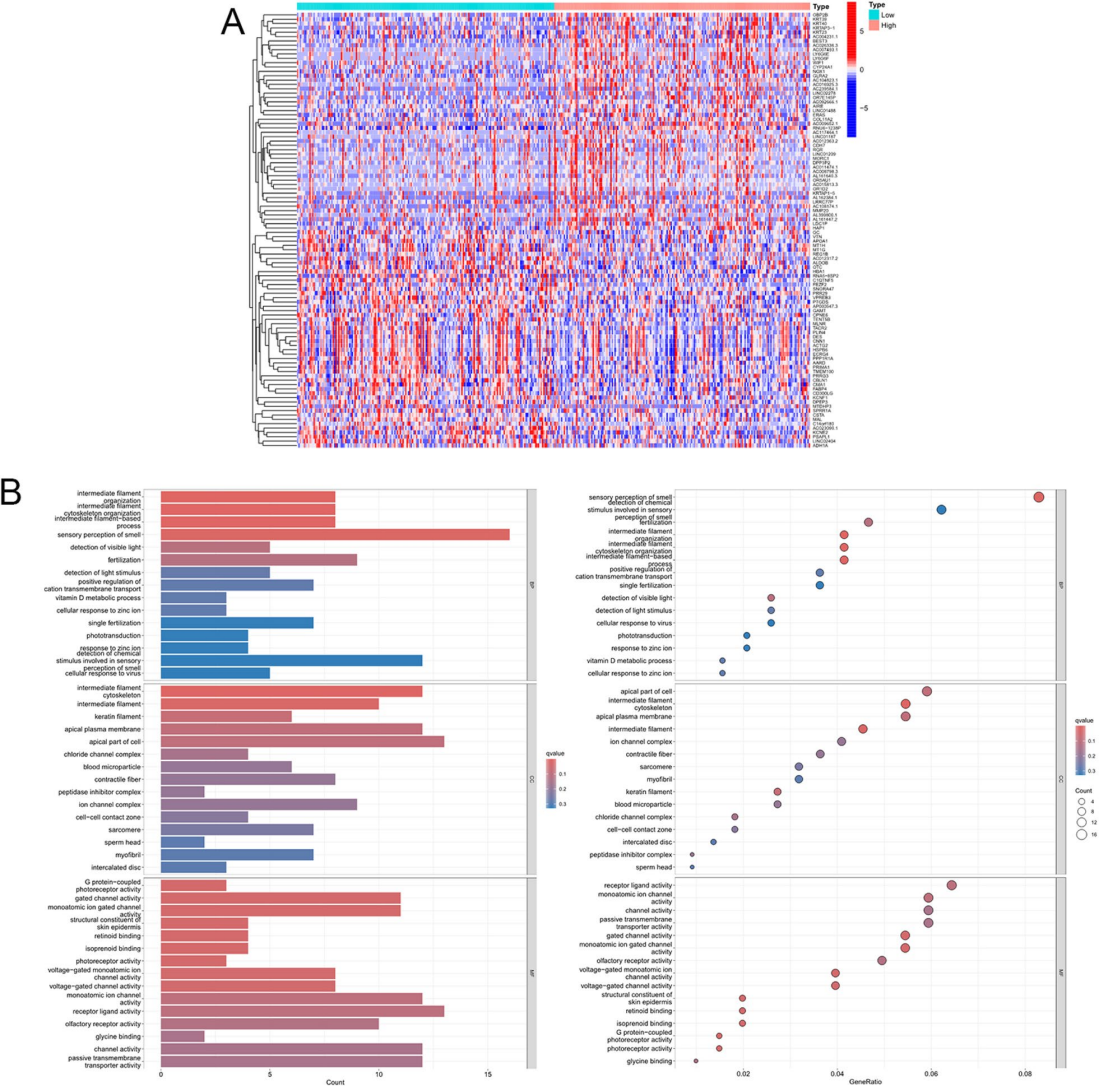

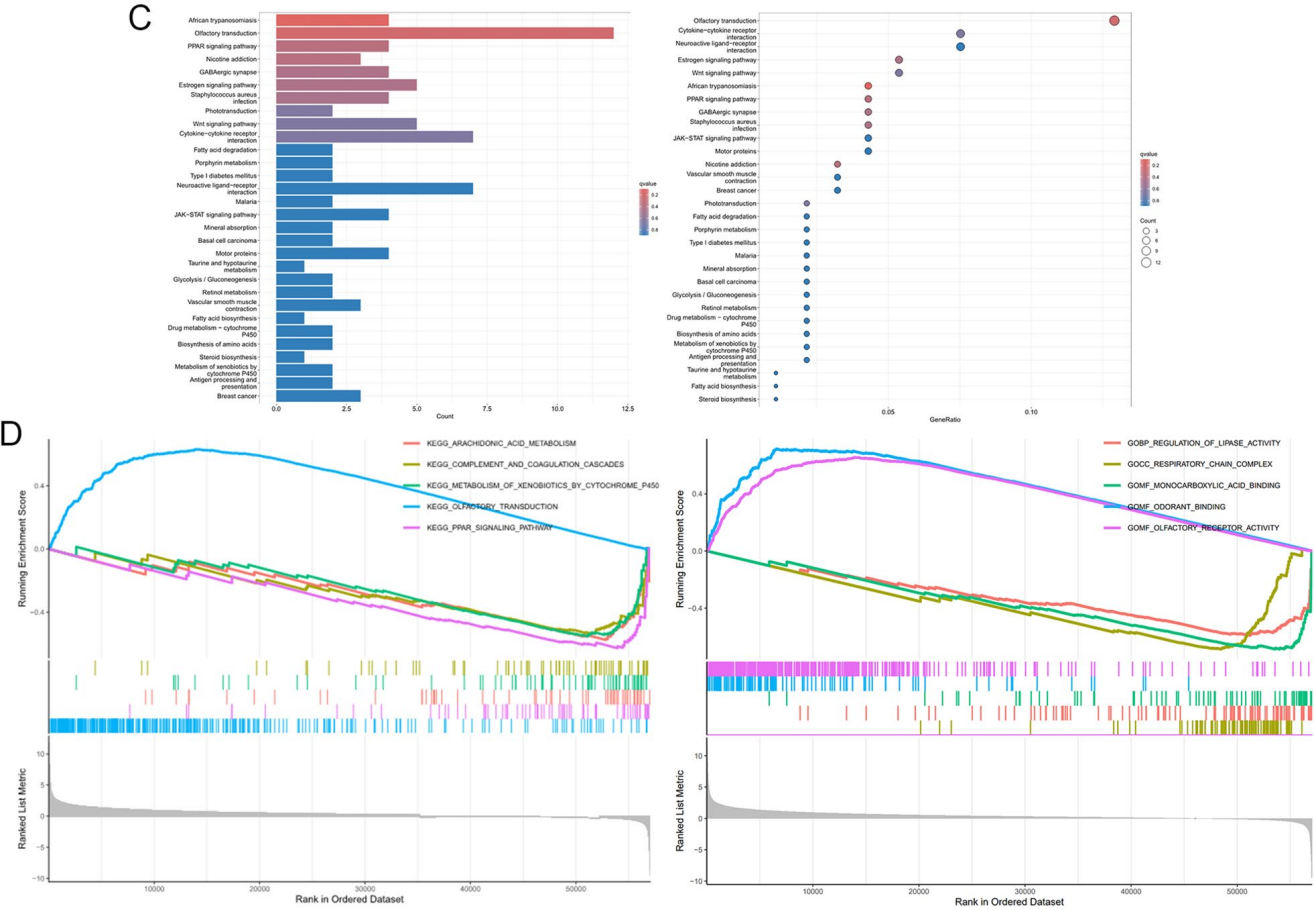
Differentially expressed genes and enrichment analysis associated with FMR1. **A** Differentially expressed genes correlated with FMR1 expression. **B** GO enrichment analysis of FMR1-associated genes. **C** KEGG pathway enrichment analysis of FMR1-associated genes. **D** GSEA enrichment analysis of FMR1-associated genes

We performed differential expression analysis stratified by FMR1 expression levels and identified the top 100 positively and negatively correlated genes (Fig. 3A). Gene Ontology (GO) enrichment analysis highlighted biological processes such as “intermediate filament cytoskeleton organization,” “keratin filament,” “apical part of cell,” “gated channel activity,” “structural constituent of skin epidermis,” and “isoprenoid binding” (Fig. 3B). KEGG pathway analysis revealed enrichment in “olfactory transduction,” “African trypanosomiasis,” and “PPAR signaling pathway,” while pathways such as “GABAergic synapse” and “JAK-STAT signaling” were less prominently enriched (Fig. 3C). In the GSEA enrichment analysis, when the enriched gene sets are from the KEGG database, it shows that the “Olfactory transduction pathway” is active in the high-expression group of the target genes, and “Arachidonic acid metabolism”, “Complement and coagulation cascades”, “Metabolism of xenobiotics by cytochrome P450”, and “PPAR signaling pathway” are active in the low-expression group of the target genes; when the enriched gene sets are from the GO database, it shows that functions such as “Odorant binding” and “Olfactory receptor activity” are active in the high-expression group of the target genes, and functions such as “Regulation of lipase”, “Respiratory chain complex”, and “Monocarboxylic acid binding” are active in the low-expression group of the target genes (Fig. 3D). These findings suggest that FMR1 and its associated genes may regulate multiple cellular pathways relevant to gastric cancer biology.

### Association of FMR1 expression with immune infiltration in gastric cancer

**Fig. 4:**
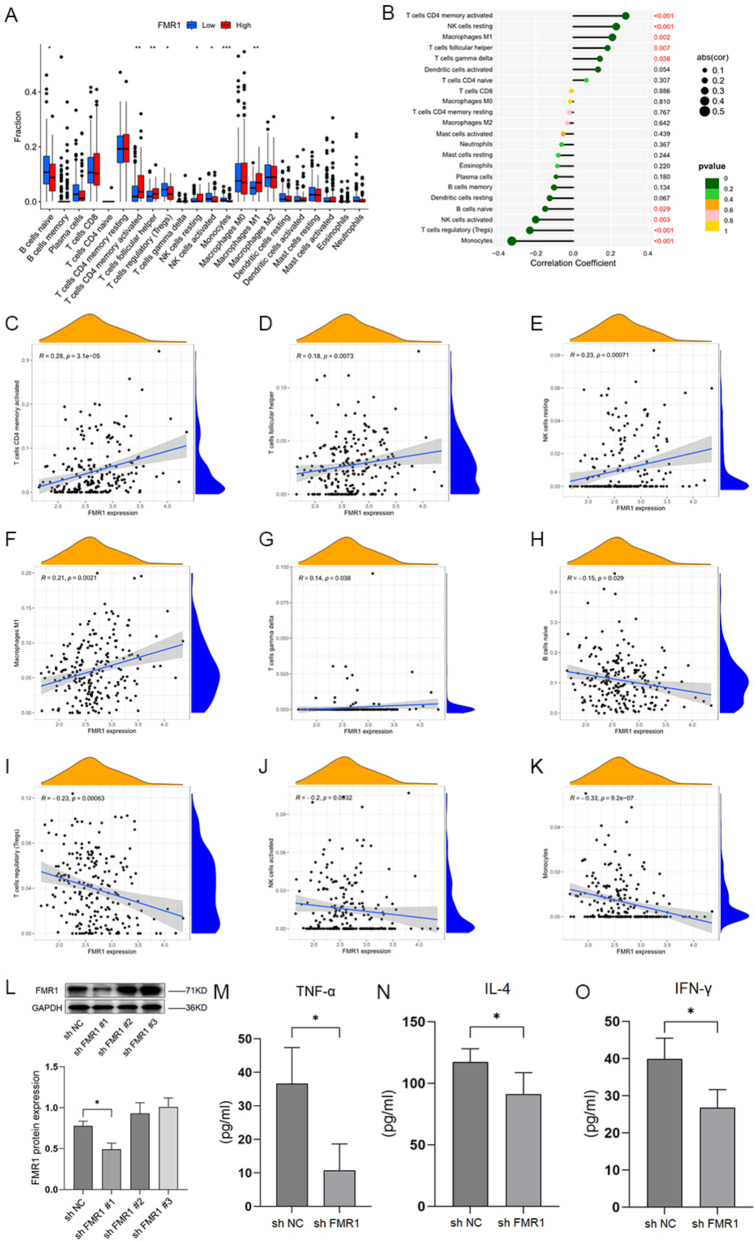
Correlation between FMR1 expression and immune infiltration in gastric cancer.** A** Boxplot showing the correlation between FMR1 expression and the abundance of 22 immune cell types in gastric cancer. **B** Bar plot showing the correlation between FMR1 expression and the abundance of 22 immune cell types in gastric cancer. **C** Correlation between FMR1 expression and activated CD4 + memory T cells. **D** Correlation between FMR1 expression and follicular helper T cells. **E** Correlation between FMR1 expression and resting NK cells. **F** Correlation between FMR1 expression and M1 macrophages. **G** Correlation between FMR1 expression and γ-δT cells. **H** Correlation between FMR1 expression and naïve B cells. **I** Correlation between FMR1 expression and regulatory T cells (Tregs). **J** Correlation between FMR1 expression and activated NK cells. **K** Correlation between FMR1 expression and monocytes. **L** Expression of FMR1 protein after transfecting gastric cancer cells with sh NC and sh FMR1. **M** TNF-α secretion level of CD4⁺ T cells after FMR1 knockdown. **N** IL-4 secretion level of CD4⁺ T cells after FMR1 knockdown. **O** IL-4 secretion level of CD4⁺ T cells after FMR1 knockdown

Correlation analyses demonstrated significant associations between FMR1 expression and the infiltration levels of various immune cells, including naïve B cells, activated CD4 + memory T cells, follicular helper T cells, regulatory T cells (Tregs), resting and activated natural killer (NK) cells, monocytes, and M1 macrophages (*P* < 0.05; Fig. 4A,B). Scatterplot analyses confirmed positive correlations between FMR1 expression and activated CD4 + memory T cells (R = 0.28, *P* = 3.1e−05), follicular helper T cells (R = 0.18, *P* = 0.0073), resting NK cells (R = 0.23, *P* = 0.0071), and M1 macrophages (R = 0.21, *P* = 0.0021) (Fig. 4C–F). Negative correlations were observed with γδ T cells (R = 0.14, *P* = 0.038), naïve B cells (R =  − 0.15, *P* = 0.029), Tregs (R = − 0.23, *P* = 0.0063), activated NK cells (R =  − 0.20, *P* = 0.0032), and monocytes (R = − 0.33, *P* = 9.2e − 07) (Fig. 4G–K). Notably, high FMR1 expression was positively correlated with immune cells such as CD4⁺ memory T cells and M1 macrophages, but negatively correlated with Tregs and monocytes. These two groups of immune cells exert opposing functions within the tumor immune microenvironment, and the consistency of our findings suggests that FMR1 may be involved in shaping the immune infiltration landscape of gastric cancer, thereby influencing immune balance and tumor progression.

To provide functional evidence for this finding, we further validated the interaction between FMR1 in gastric cancer cells and CD4⁺ T cells through in vitro assays. As a major immune component of the tumor microenvironment, the response of CD4⁺ T cells serves as an indicator of the host immune reaction to tumor cells [21]. We established gastric cancer cell lines with stable FMR1 knockdown via lentiviral transduction and found that shFMR1#1 markedly reduced FMR1 expression (Fig. 4L); thus, shFMR1#1 was selected for subsequent experiments. SNU-484 cells with FMR1 knockdown or control cells were co-cultured with human CD4⁺ T cells, and cytokine levels in the supernatants were measured by ELISA. The results showed that after 24 h of co-culture, TNF-α and IL-4 levels in the shFMR1 group were significantly lower than in the control group (Fig. 4M,N); after 48 h, IFN-γ levels were also markedly reduced (Fig. 4O). These findings indicate that knockdown of FMR1 in tumor cells suppresses the secretion of TNF-α, IFN-γ, and IL-4 by CD4⁺ T cells, thereby attenuating their activation state.

### Single-cell transcriptomic analysis reveals cellular heterogeneity in gastric cancer

**Fig. 5:**
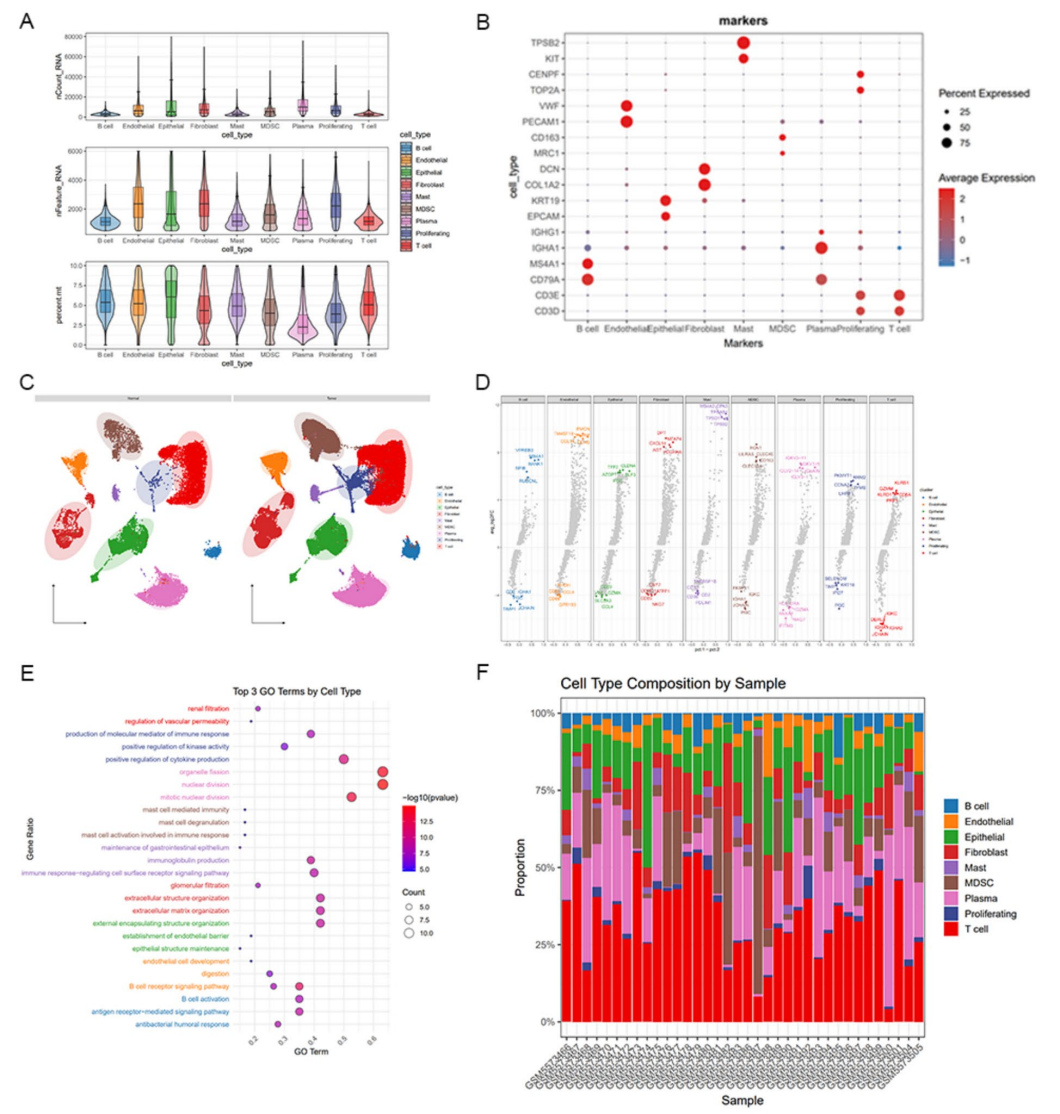
Single-cell transcriptomic analysis revealing cellular composition and functional heterogeneity in gastric cancer samples.** A** Display of quality control results. **B** Cell clustering results based on highly variable genes using PCA and UMAP dimensionality reduction. **C** Dot plot of marker gene expression across annotated cell types. **D** The top 5 upregulated and downregulated specifically differentially expressed genes in each cell type. **E** Bubble plot of GO enrichment analysis for major cell types. **F** Statistical chart of the proportion of each cell type in tumor samples and normal samples

We analyzed single-cell RNA-seq data from 36 gastric samples (26 tumor and 10 paired normal tissues), generating a comprehensive atlas of 117,965 high-quality cells (Quality control standards: remove low-quality cells with a mitochondrial gene ratio > 10%, the number of expressed genes < 200 or > 5000), it reveals the significant differences in cell composition between tumors and normal tissues. Standardized quality control and unsupervised cluster analysis identified 25 biological subpopulations (Fig. 5A). Based on canonical marker genes [22] (Fig. 5B), nine major cell types were annotated: T cells (CD3D/CD3E), B cells (CD79A/MS4A1), plasma cells (CD79A/MS4A1), epithelial cells (EPCAM/KRT19), fibroblasts (COL1A2/DCN), myeloid cells (MRC1/CD163), endothelial cells (PECAM1/VWF), proliferating cells (TOP2A/CENPF), and mast cells (KIT/TPSB2). Dot plots demonstrated consistent expression patterns supporting accurate annotations (Fig. 5C). Differential expression analysis (Fig. 5D) revealed cell type–specific transcriptional signatures, while the heatmap of the top 5 differentially expressed genes provides a molecular basis for functional analysis. GO enrichment analysis indicated fibroblasts were enriched for extracellular matrix remodeling and collagen assembly, epithelial cells for polarity and barrier functions, T cells for immune synapse formation and chemokine signaling, and B cells for BCR signaling and antibody secretion (Fig. 5E). The reliability of the annotation system has been verified from the functional level. Quantitative analysis of cell composition showed immune cells accounted for 58.6% of the tumor microenvironment (TME), with T cells (32.1%) and myeloid cells (15.4%) as dominant subsets (Fig. 5F), reveal the significant heterogeneity of the immune microenvironment in gastric cancer. This discovery provides crucial data support for deciphering the mechanism of tumor immune escape and developing targeted immunotherapy strategies.

### Identification of malignant epithelial cells and tumor-specific FMR1 expression patterns

**Fig. 6:**
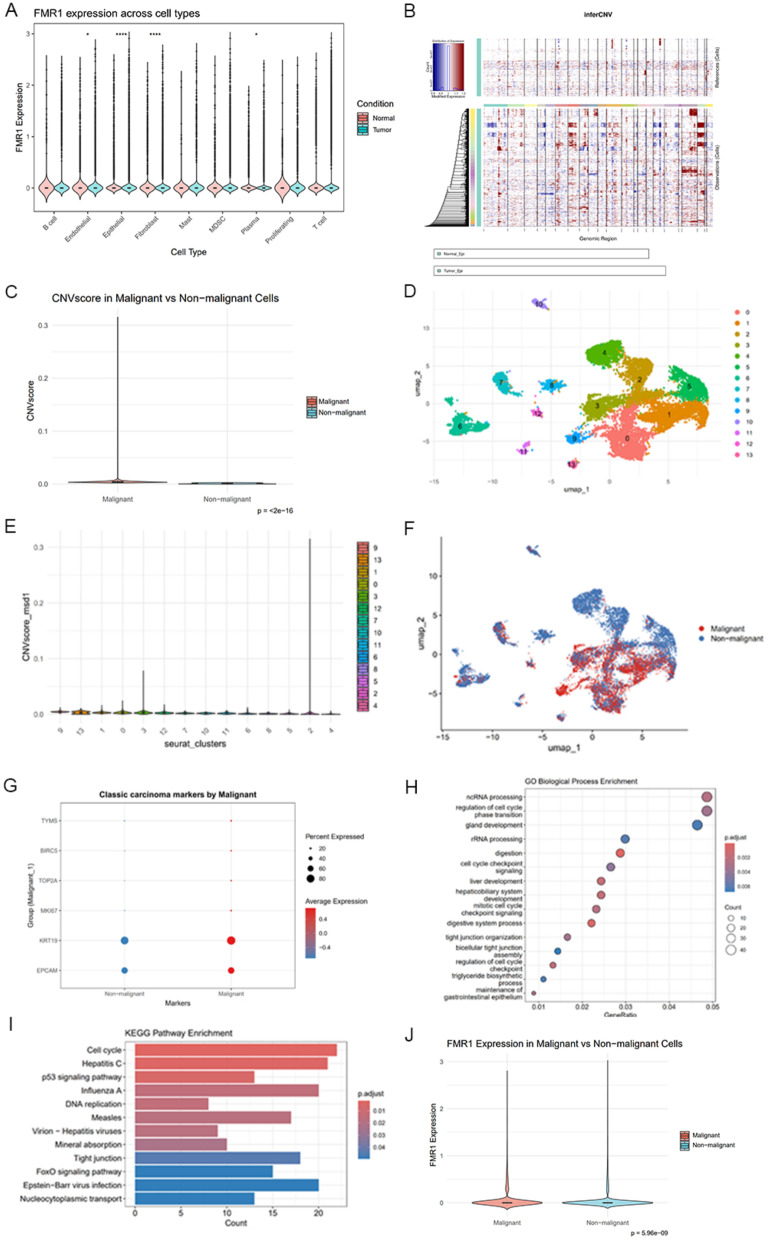
Identification of malignant epithelial cells and FMR1 expression characteristics.** A** FMR1 expression levels across different cell types. **B** CNV heatmap generated from inferCNV analysis. **C** Comparison of CNV scores between malignant and non-malignant epithelial cells. **D** UMAP dimensionality reduction plot of epithelial cells. **E** Distribution of CNV scores across 15 epithelial cell subclusters. **F** UMAP visualization of epithelial cells stratified into malignant and non-malignant groups based on CNV scores. **G** Expression of classic cancer-related markers in malignant and non-malignant epithelial cells. **H** GO enrichment analysis of differentially expressed genes in malignant cells versus non—malignant cells. **I** KEGG enrichment analysis of differentially expressed genes in malignant cells versus non—malignant cells. **J** Comparison of FMR1 expression levels between malignant and non-malignant epithelial cells

To systematically analyze the characteristics of malignant transformation of gastric epithelial cells (ECs) in the system, we first examined FMR1 expression across cell types. FMR1 was selectively upregulated in tumor-derived epithelial cells (Fig. 6A), suggesting that it may play a key regulatory role in the occurrence and development of gastric cancer. Considering that there may still be paracancerous cells or cell populations that have not been fully transformed in the tumor tissue, to further clarify whether FMR1 is expressed in malignant epithelial cells, we further performed copy number variation (CNV) analysis using the inferCNV algorithm (v1. 20.0). Using the 90th percentile of CNVscore in the reference group (normal epithelium) as the threshold [12], cells with CNVscore above this cutoff were defined as malignant epithelial cells (malignant ECs), whereas the remainder were defined as non-malignant ECs (Fig. 6B). Comparison of CNVscores revealed that malignant ECs displayed significantly higher scores than non-malignant ECs (*p* < 2 × 10^ − 16; Fig. 6C). We further reclustered epithelial cells into 14 subclusters and visualized the groups using UMAP. Analysis of CNVscore distribution across epithelial subclusters showed that cluster 9 exhibited the highest score (Fig. 6D–F). In addition, we collected commonly reported malignancy markers from the literature: KI67, TOP2A, TYMS, BIRC5, EPCAM, and KRT19 [23–28]. Multiple malignancy-related epithelial proliferation markers were significantly higher overall in malignant ECs than in the non-malignant cell group (Fig. 6G), further supporting the validity of CNV-based classification. On this basis, we compared differentially expressed genes between malignant and non-malignant ECs and performed GO and KEGG enrichment analyses. GO biological processes were significantly enriched in maintenance in gastrointestinal epithelium, bicellular tight junction assembly, cell cycle checkpoint signaling, rRNA/ncRNA processing, and gland development (Fig. 6H), suggesting that malignant ECs are characterized by both epithelial barrier remodeling and enhanced cell cycle programs. KEGG pathways were significantly enriched in cell cycle, DNA replication, p53 signaling pathway, and tight junctions (F6g. 6I), consistent with the GO results. Notably, FMR1 expression was significantly upregulated in malignant ECs compared with non-malignant ECs (Fig. 6J), suggesting that FMR1 may contribute to malignant evolution of gastric cancer, potentially through processes affecting genome stability.

### Biological functions of malignant cell populations with high FMR1 expression

**Fig. 7:**
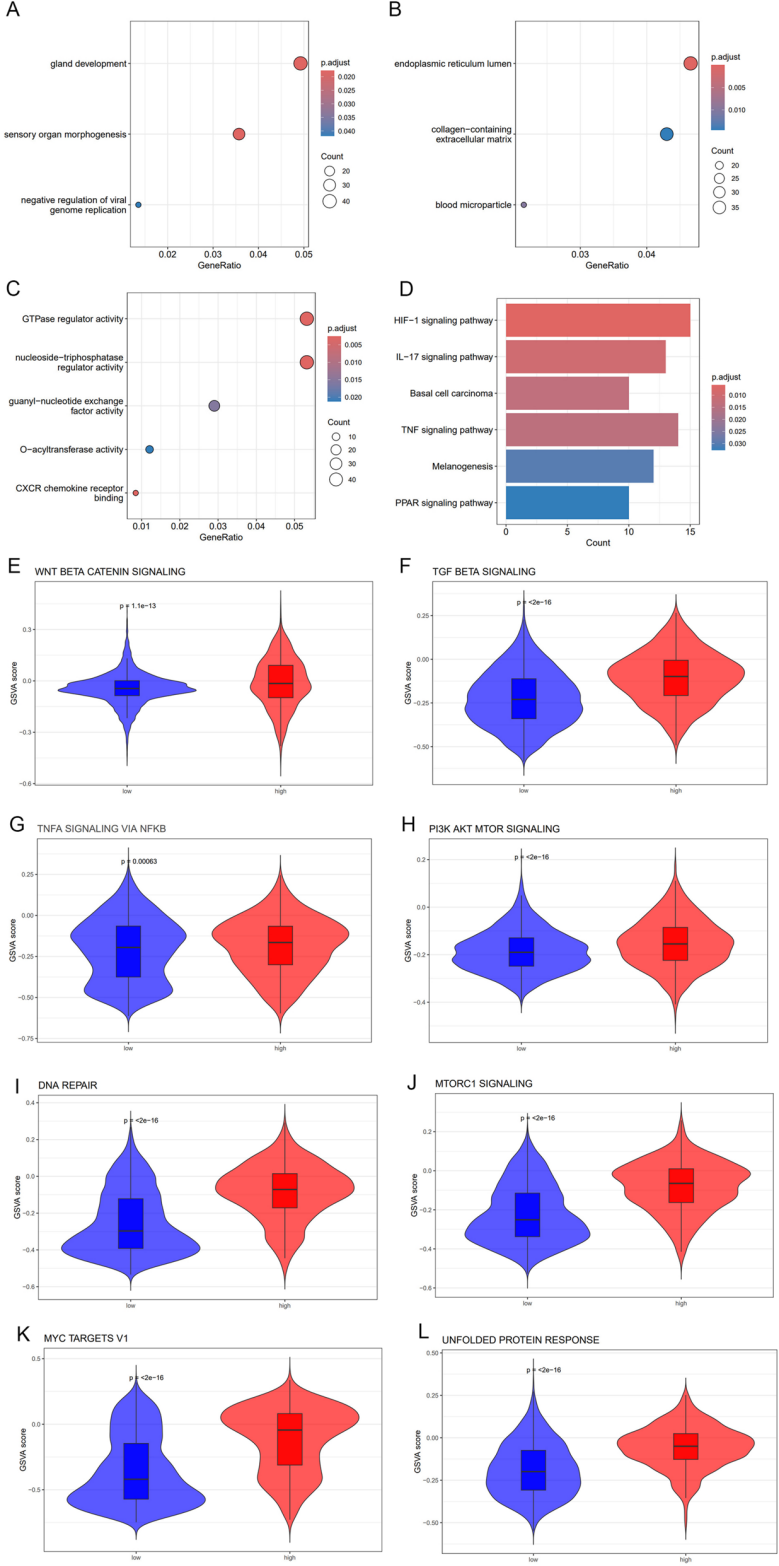
Functional pathway analysis of high and low FMR1 expression groups in malignant epithelial cells. **A** Enrichment of differentially expressed genes in the high and low FMR1 groups of malignant cells in GO-BP.** B** Enrichment of differentially expressed genes in the high and low FMR1 groups of malignant cells in GO-CC.** C** Enrichment of differentially expressed genes in the high and low FMR1 groups of malignant cells in GO-MF. **D** Enrichment of differentially expressed genes in the high and low FMR1 groups of malignant cells in KEGG. **E** Activity of the HALLMARK_WNT_BETA_CATENIN_SIGNALING pathway in malignant epithelial cells with high FMR1 expression. **F** Activity of the HALLMARK_TGF_BETA_SIGNALING pathway in malignant epithelial cells with high FMR1 expression. **G** Activity of the HALLMARK_TNFA_SIGNALING_VIA_NFKB signaling pathway in malignant epithelial cells with high FMR1 expression. **H** Activity of the HALLMARK_PI3K_AKT_MTOR_SIGNALING pathway in malignant epithelial cells with high FMR1 expression. **I** Activity of the HALLMARK_DNA_REPAIR signaling pathway in malignant epithelial cells with high FMR1 expression. **J** Activity of the HALLMARK_MTORC1_SIGNALING pathway in malignant epithelial cells with high FMR1 expression. **K** Activity of the HALLMARK_MYC_TARGETS_V1 signaling pathway in malignant epithelial cells with high FMR1 expression. **L** Activity of the HALLMARK_UNFOLDED_PROTEIN_RESPONSE signaling pathway in malignant epithelial cells with high FMR1 expression

To delineate the functional characteristics of malignant epithelial cells (ECs) with high FMR1 expression, malignant ECs were stratified into high and low groups based on the median level of FMR1 expression. Differential gene enrichment analysis and GSVA were performed, and group differences were quantitatively illustrated using dot plots, bar plots, and violin plots. GO enrichment analysis of differentially expressed genes, especially in the GO-BP, revealed that FMR1-high cells were significantly associated with sensory organ morphogenesis, gland development, and negative regulation of viral genome replication (Fig. 7A–C). KEGG pathway analysis further highlighted tumor- and inflammation/hypoxia-related pathways, including TNF signaling pathway, IL-17 signaling pathway, HIF-1 signaling pathway, basal cell carcinoma, and PPAR signaling pathway (Fig. 7D), suggesting that this population is involved not only in proliferation but also in the reprogramming of inflammatory and stress-response networks. We next performed GSVA based on the expression matrix of FMR1-high cells to evaluate pathway activity (Additional file 1). Stemness-, chemoresistance-, and immune evasion–related gene sets were specifically examined, and these pathways were consistently and significantly enriched in malignant epithelial cells with high expression of FMR1 (FDR ≤ 0.05) (Fig. 7E–L). Overall, this subpopulation exhibited enhanced stemness, elevated DNA repair and proteostasis stress, and activation of PI3K–AKT–mTOR and TNFα–NF-κB anti-apoptotic signaling together with TGF-β–mediated immune exclusion, constituting a combined molecular signature of “enhanced stemness, chemoresistance, and an immunosuppressive microenvironmental tendency.”

### Knockdown of FMR1 suppresses gastric cancer cell proliferation, migration, and invasion

**Fig. 8:**
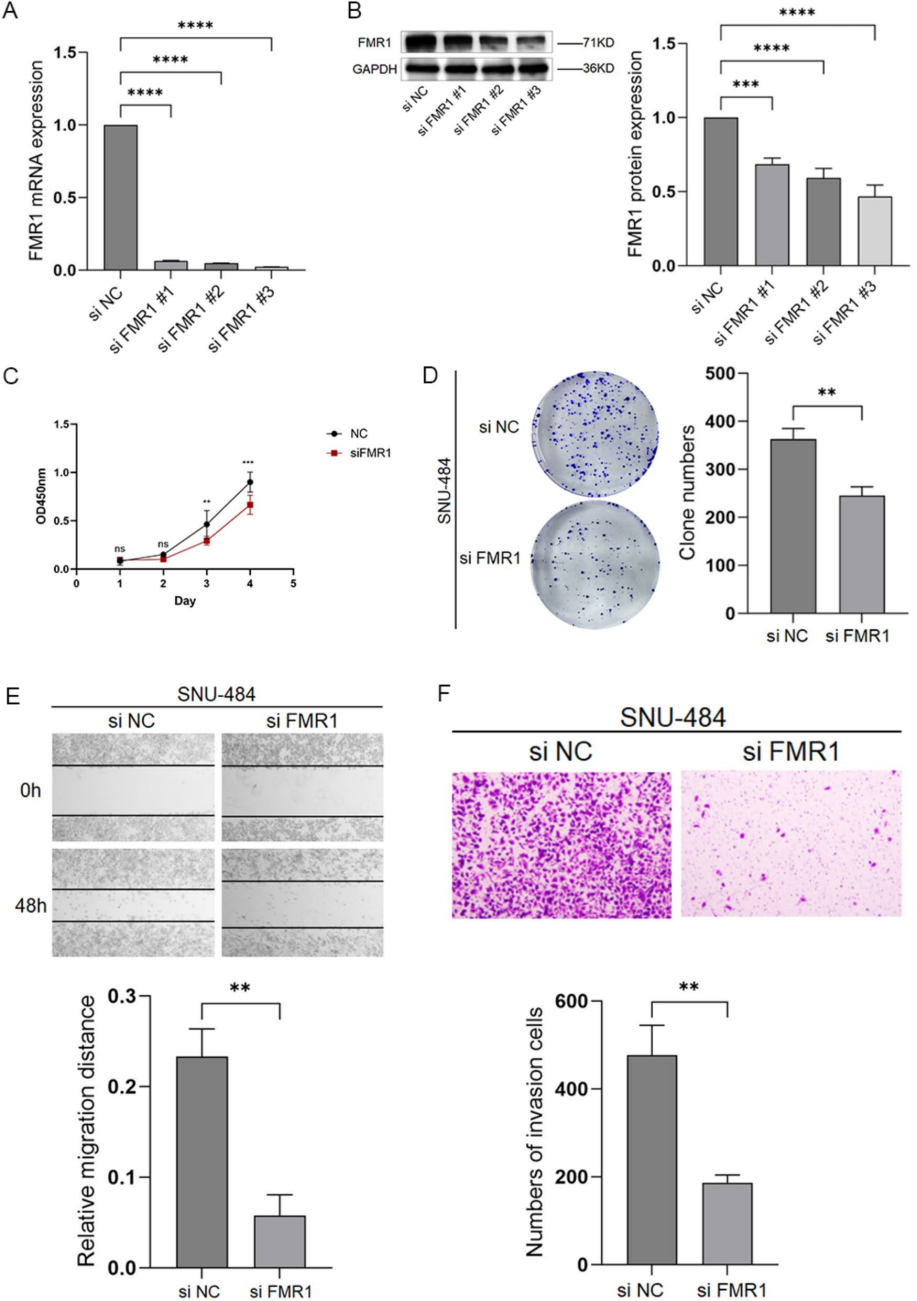
Knockdown of FMR1 in gastric cancer cells. **A** FMR1 mRNA expression in gastric cancer cells transfected with siNC or siFMR1. **B** FMR1 protein expression in gastric cancer cells transfected with siNC or siFMR1. **C** CCK-8 assay measuring proliferation of SNU-484 cells. **D** Colony formation assay measuring proliferation of SNU-484 cells. **E** Wound healing assay assessing migration capability. **F** Transwell invasion assay assessing invasive capability

As shown above, the expression level of FMR1 protein is relatively high in some gastric cancer cell lines, especially in SNU-484, this line was selected for functional studies. siRNA-mediated knockdown significantly reduced FMR1 mRNA and protein levels, with siFMR1 #3 achieving the greatest silencing (Fig. 8A,B), it will continue to be used in subsequent experiments. To investigate the effect of FMR1 on the proliferation ability of gastric cancer cells, we demonstrated through the CCK—8 proliferation assay (Fig. 8C) and the plate colony formation assay (Fig. 8D) that, compared with control cells, FMR1 knockdown inhibited the proliferation ability of gastric cancer cells. To study the effect of FMR1 on the motility of gastric cancer cells, we proved via the wound—healing assay (Fig. 8E) and the Transwell invasion assay (Fig. 8F) that, compared with control cells, FMR1 knockdown inhibited the migration and invasion abilities of gastric cancer cells.

#### FMR1 regulates c-MYC expression in gastric cancer cells

**Fig. 9:**
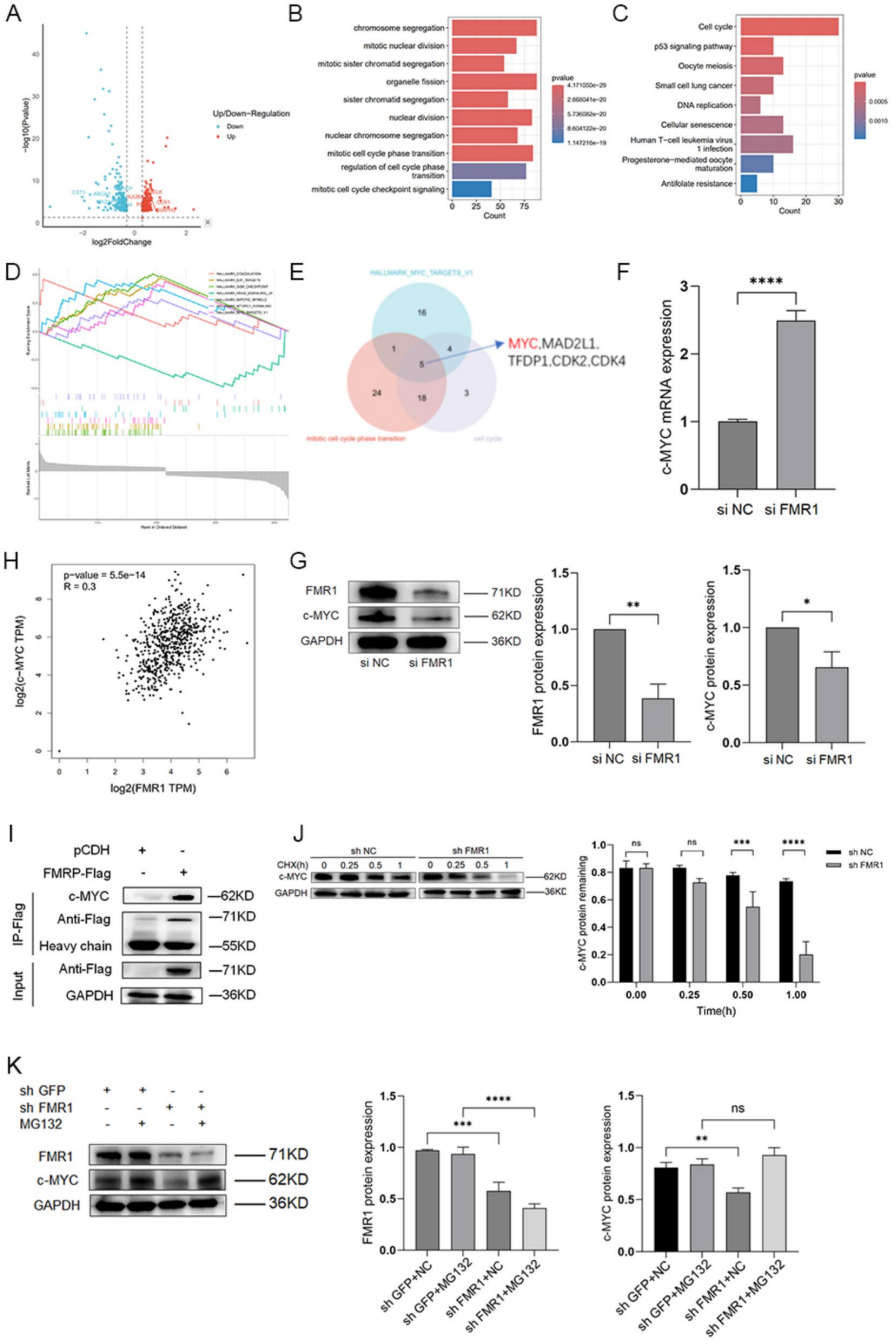
Regulation of c-MYC by FMR1 in human gastric cancer cells. **A** Volcano plot of differentially expressed genes between FMR1 knockdown and control groups.** B** GO enrichment classification of differentially expressed genes. **C** KEGG pathway enrichment classification of differentially expressed genes. **D** GSEA enrichment analysis of differentially expressed genes. **E** Identification of c-MYC as a key gene based on enrichment analysis. **F** qRT-PCR showing upregulation of c-MYC mRNA upon FMR1 silencing in SNU-484 cells. **G** Western blot showing downregulation of c-MYC protein upon FMR1 silencing in SNU-484 cells. **H** Analysis of the expression correlation between FMR1 and c-MYC. **I** Verification of the protein interaction between FMRP and c-MYC. **J** FMR1 knockdown promotes the degradation of c-MYC protein in gastric cancer cells. **K** The proteasome inhibitor MG132 partially reverses the downregulation of c-MYC protein caused by FMR1 knockdown

To elucidate downstream effectors of FMR1, high-throughput transcriptome sequencing of mRNA (high throughout transcriptome sequencing of mRNA, mRNA ⁃seq) was performed on SNU-484 siFMR1 cells with FMR1 knocked down and negative control SNU-484 NC cells to identify differentially expressed genes. The sequencing results showed that 424 differentially expressed genes were obtained after knocking down FMR1 (Additional file 2, Fig. 9A). KEGG, GO, and GSEA analyses highlighted tumor-related pathways such as “HALLMARK_MYC_TARGETS_V1,” “Cell cycle,” and “Mitotic cell cycle phase transition” (Fig. 9B–D). A Venn diagram was drawn for the genes included in the above gene sets and pathways to obtain the intersection, among which c-MYC caught our attention (Fig. 9E). We further examined the effect of FMR1 on the expression of c-MYC, found that with the knockdown of FMR1, the mRNA expression of c-MYC increased (Fig. 9F), which was consistent with the RNA-seq results. The protein expression of c-MYC decreased (Fig. 9G), which was consistent with the change in FMR1 protein expression. Correlation analysis using GEPIA (http://gepia.cancer-pku.cn/) further supported a positive relationship between FMR1 and c-MYC expression in gastric cancer (R = 3; *P* = 5.5e−14; Fig. 9H).

#### FMR1 stabilizes c-MYC protein by suppressing proteasomal degradation

As described above, c-MYC protein and mRNA levels exhibited discordant changes upon FMR1 knockdown, suggesting that FMR1 primarily regulates c-MYC at the post-transcriptional level. Exogenous co-immunoprecipitation assays confirmed a direct protein–protein interaction between FMR1 and c-MYC (F9g. 9I). We then blocked protein synthesis with cycloheximide, and the results showed that FMR1 knockdown markedly accelerated c-MYC degradation in gastric cancer cells (Fig. 9J). It is well established that protein degradation in eukaryotic cells mainly occurs through two major pathways: the proteasome and the lysosome[29]. Moreover, c-MYC has a short protein half-life, and its abundance is tightly controlled by the ubiquitin–proteasome pathway [30]. To validate this, we further employed the proteasome inhibitor MG132, and found that the c-MYC downregulation induced by FMR1 knockdown was significantly reversed by MG132 (Fig. 9K).

#### FMR1 promotes gastric cancer cell proliferation, migration, and invasion via c-MYC

**Fig. 10:**
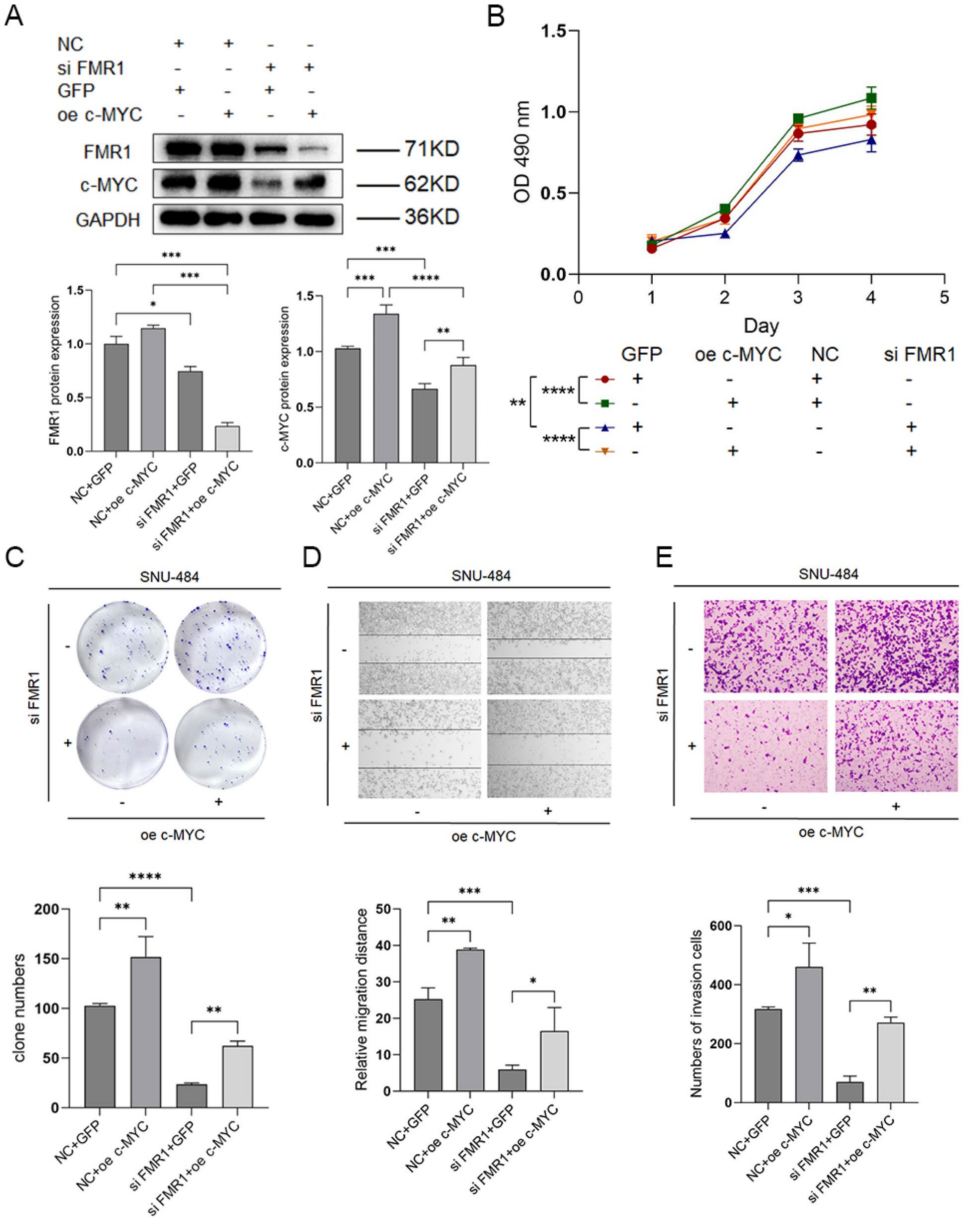
Effects of c-MYC overexpression on reversing FMR1 knockdown in gastric cancer cells. **A** Detection of FMR1 and c-MYC protein expression in SNU-484 cells with or without FMR1 knockdown and c-MYC overexpression. B. CCK-8 assay of SNU-484 cells with or without FMR1 knockdown and c-MYC overexpression. **C** Colony formation assay of SNU-484 cells with or without FMR1 knockdown and c-MYC overexpression. **D** Wound healing assay of SNU-484 cells with or without FMR1 knockdown and c-MYC overexpression. **E** Transwell invasion assay of SNU-484 cells with or without FMR1 knockdown and c-MYC overexpression

**Fig. 11:**
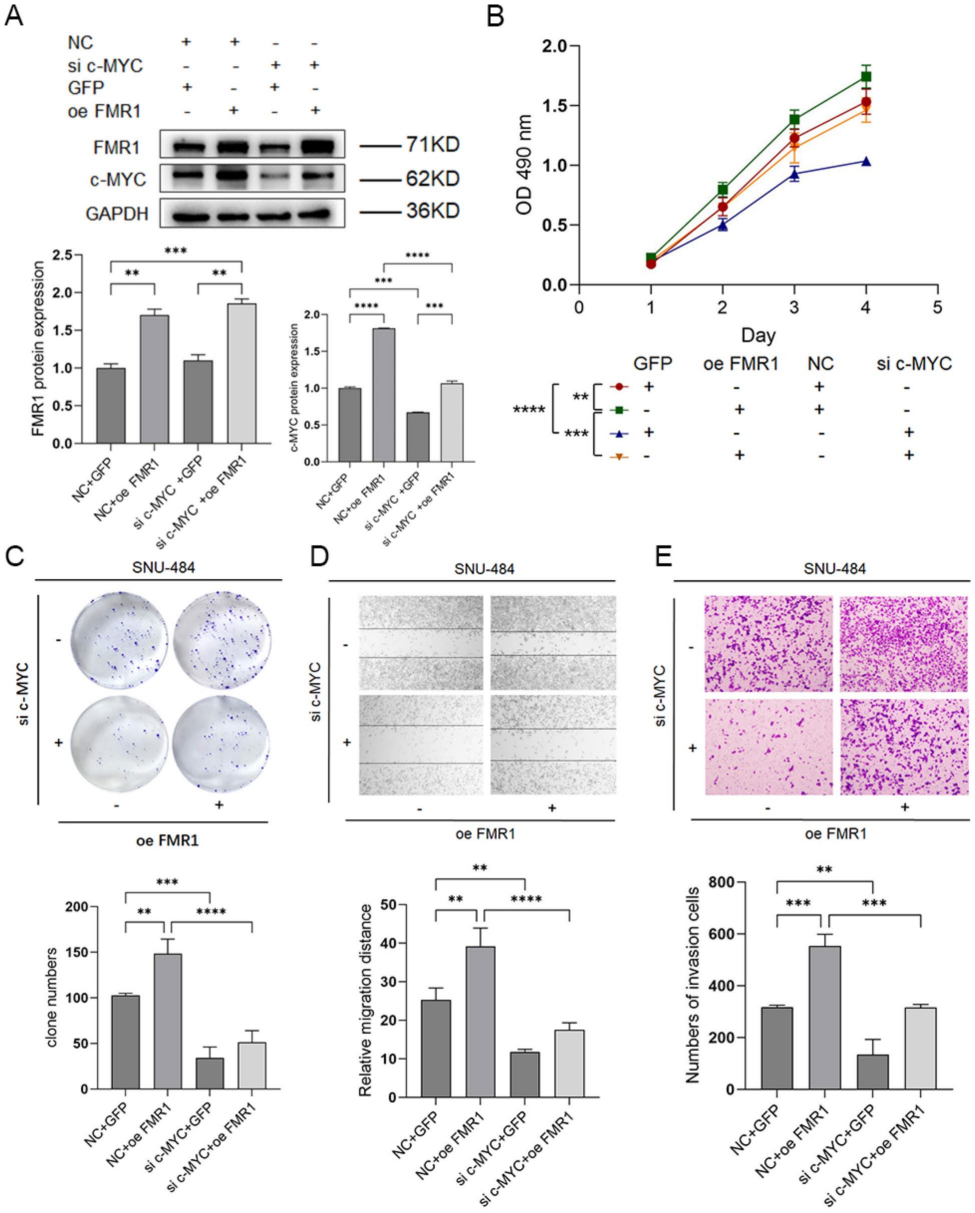
Effects of c-MYC knockdown on reversing FMR1 overexpression in gastric cancer cells. **A** Detection of FMR1 and c-MYC protein expression in SNU-484 cells with or without FMR1 overexpression and c-MYC knockdown. **B** CCK-8 assay of SNU-484 cells with or without FMR1 overexpression and c-MYC knockdown. **C** Colony formation assay of SNU-484 cells with or without FMR1 overexpression and c-MYC knockdown. **D** Wound healing assay of SNU-484 cells with or without FMR1 overexpression and c-MYC knockdown. **E** Transwell invasion assay of SNU-484 cells with or without FMR1 overexpression and c-MYC knockdown

To determine whether FMR1 exerts its oncogenic effects through c-MYC, we performed rescue experiments. Knockdown of FMR1 decreased both FMRP and c-MYC protein levels, while forced c-MYC overexpression restored c-MYC expression (Fig. 10A). Functionally, FMR1 knockdown significantly inhibited proliferation, colony formation, migration, and invasion, effects that were partially reversed by c-MYC overexpression (Fig. 10B–E). Conversely, overexpression of FMR1 increased FMRP and c-MYC levels, after knocking down the c-MYC gene, the expression of c-MYC protein decreased accordingly (Fig. 11A), attenuating FMR1-driven enhancements in proliferation, colony formation, migration, and invasion (Fig. 11B–E). These results indicate that c-MYC is a critical mediator of FMR1’s oncogenic activity in gastric cancer.

## Discussion

FMR1 was originally recognized for its association with the neurodevelopmental disorder fragile X syndrome (FXS) and has traditionally been studied in the context of the central nervous system [31]. It is involved in multiple forms of synaptic regulation, including synaptic plasticity [32] and the maintenance of synaptic homeostasis [33], and loss of FMRP leads to synaptic dysfunction and consequent cognitive [34] and behavioral deficits. Although the neuronal functions of FMRP have been extensively described, growing evidence indicates that FMRP exerts important roles beyond neurons and displays multifaceted biological properties. Earlier studies reported reduced incidence of various cancer types in two independent FXS patient cohorts from Denmark and the United Kingdom, and a case report further described abnormally attenuated glioblastoma growth in an FXS boy [35].

Growing evidence indicates that FMR1 plays a pivotal role in the initiation and progression of solid tumors, driving proliferation, differentiation, invasion, and metastasis througha variety of complex molecular regulatory networks and signal transduction pathways, closely related to tumor progression, drug resistance, and the prognosis of patients. Preclinical studies on using the FMR1 gene as a target to guide tumor treatment have currently achieved some preliminary results. In this study, through a comprehensive analysis of the expression of FMR1 in gastric cancer, its clinicopathological features, immune infiltration, and its impact on the biological functions of gastric cancer cells, the important role of FMR1 in the occurrence and development of gastric cancer was revealed. Our research results show that FMR1 is not only highly expressed in gastric cancer tissues and cell lines but also closely related to the poor prognosis of patients, clinicopathological features, and immune cell infiltration. By knocking down FMR1, the proliferation, migration, and invasion abilities of gastric cancer cells were inhibited. In addition, RNA-seq, IP–Western blotting, cycloheximide chase, proteasome inhibition, and functional rescue assays were also demonstrated that FMR1 significantly affects the biological behavior of gastric cancer cells by regulating the c—MYC pathway, further confirming the oncogenic role of FMR1 in gastric cancer.

First, we found that FMR1 was upregulated in gastric cancer, which was consistent with previous research findings [36]. Moreover, the overexpression of FMR1 may promote the proliferation and survival of cancer cells by regulating the cell cycle, DNA repair, and anti—apoptotic pathways [5, 37]. Our pan-cancer analysis further revealed that FMR1 exhibits differential expression patterns across cancer types. In addition to gastric cancer, FMR1 was markedly upregulated in cancers such as cholangiocarcinoma (CHOL) and colon adenocarcinoma (COAD), whereas it was downregulated in cancers including glioblastoma multiforme (GBM), kidney renal clear cell carcinoma (KIRC), and prostate adenocarcinoma (PRAD), among others.. This divergence suggests that the role of FMR1 is strongly tissue-specific. Moreover, the functions of FMR1 vary across tumor stages and anatomical sites, consistent with its heterogeneous expression in different human tissues [38]. As an RNA-binding protein, FMR1 exerts functions that depend on cellular context and downstream targets, and may regulate distinct transcriptional or immune-related pathways across tissues. For example, Antonio Di Grazia et al. [39] reported that fragile X mental retardation protein (FMRP), an RBP, is involved in multiple steps of mRNA metabolism and modulates mRNA stability, transport, or editing according to the properties of its targets and the cellular environment. Thus, FMRP may exert profoundly different functions in different tissues through diverse downstream targets and regulatory pathways. Using TCGA data, we confirmed higher FMR1 expression in gastric cancer compared with normal gastric tissues, a finding validated by qRT-PCR, Western blotting, and immunohistochemistry. These results highlight FMR1 as a potential diagnostic biomarker for distinguishing malignant from normal gastric tissues, can be used for the early diagnosis of gastric cancer. In—depth survival analysis and prognostic evaluation show that FMR1 has the potential to become a molecular diagnostic marker for poor prognosis in gastric cancer patients. In addition, the analysis of public databases and clinical data also jointly proves that the expression level of FMR1 in gastric cancer tissues is significantly correlated with the TNM stage.

Tumor immunity plays an irreplaceable role in the origin, progression, recurrence and outcome of tumors. In recent years, treatment strategies developed based on tumor immune mechanisms, namely tumor immunotherapy, have become an emerging pillar in the fight against tumors [40]. Previous studies have demonstrated that the expression of FMR1 is closely related to the infiltration of multiple immune cells, especially the numbers of activated CD4-positive memory T cells, M1 macrophages and NK cells [38, 41]. This study also demonstrated that FMR1 expression was positively correlated with activated CD4⁺ memory T cells and M1 macrophages, but negatively correlated with Tregs and monocytes, providing new insights into tumor immunotherapy for gastric cancer patients with high FMR1 expression. By regulating immune cell infiltration, FMR1 may promote immune escape of gastric cancer, allowing tumors to survive under immune surveillance [42, 43]. High FMR1 expression correlated with reduced infiltration of naïve B cells, Tregs, activated NK cells, and monocytes—immune cell types whose abundance is often associated with better prognosis. Previous studies have also reported that tumor-infiltrating FoxP3⁺ regulatory T cells (Tregs) are associated with favorable prognosis in colorectal, head and neck, and esophageal cancers [44]. In our study, we similarly observed that high levels of Treg infiltration were correlated with better prognosis in gastric cancer, further supporting the relationship between Treg abundance and tumor outcomes. Consistent with our findings, previous research demonstrated that high densities of NK cells in gastric cancer patients are associated with improved prognosis compared to low densities [45]. In summary, targeting FMR1 may not only prolong overall survival in patients with gastric cancer but also facilitate adoptive immunotherapy. Upon activation, memory CD4⁺ T cells can rapidly and robustly secrete multiple effector cytokines, including IFN-γ, TNF-α, IL-2, and IL-4 [46]. Guided by bioinformatic predictions, we therefore co-cultured tumor cells with CD4⁺ T cells and measured cytokines in the supernatant, and the results confirmed that FMR1 promotes CD4⁺ T-cell activation. This finding suggests that FMR1 could serve as a novel immunotherapeutic target, potentially providing new avenues for gastric cancer treatment in combination with existing strategies such as immune checkpoint inhibitors. Moreover, FMR1 expression levels may represent a potential biomarker for predicting immunotherapy response, enabling patient stratification and personalized treatment. Although our study provides preliminary insights into the association between immune cell infiltration and FMR1 expression in relation to gastric cancer prognosis, further research is needed to elucidate the mechanistic roles of different immune cell subsets in gastric cancer development and progression.

Functional enrichment analyses implicated FMR1 in diverse biological processes. Notably, FMR1 in gastric cancer was associated with the “keratin filament” functional category. Epithelial cells can be classified based on their cytokeratin expression patterns, which vary widely among different epithelial cell types but typically remain stable even during malignant transformation. As a result, cytokeratin profiles have been widely used as diagnostic “fingerprints” for various cancers [47]. FMR1 was also linked to “gated channel activity” in gastric cancer, a functional category relevant to ion channels across the cell membrane. Abnormal ion channel function has been implicated in many cancers; for example, Kv2.1 is a pro-apoptotic K⁺ channel in medulloblastoma cells [48]. However, the specific biological roles of ion channels in gastric cancer remain to be fully elucidated. In addition, since its discovery in the 1990s, the PPAR signaling pathway has attracted considerable research interest. Extensive studies have established that PPAR signaling plays crucial roles in cell differentiation, inflammation, lipid and glucose metabolism, immune regulation, and tumorigenesis. Dysregulation of the PPAR pathway has been frequently associated with cancer development and progression [49]. The observed correlation between FMR1 expression and the PPAR signaling pathway in gastric cancer in this study supports this link, although the precise molecular mechanisms underlying their interaction warrant further investigation.

Based on the GSE183904 dataset, this study employed single-cell RNA sequencing to comprehensively characterize the cellular heterogeneity and functional properties within gastric cancer tissues, with a particular focus on the role of FMR1 in the malignant transformation of epithelial cells and its potential association with copy number variation (CNV). Malignant and non—malignant epithelial cells in gastric cancer tissues were successfully distinguished by CNVscore, which was supported by a variety of classic tumor markers. The results of differential gene analysis and functional enrichment showed that malignant epithelial cells were accompanied by obvious epithelial barrier remodeling and enhanced cell cycle programs, presenting features of genomic instability and malignant proliferation. Regarding the association between FMR1 and CNV, on the one hand, previous studies have shown that FMRP participates in transcription-coupled homologous recombination and the maintenance of R-loop dynamics; its loss leads to R-loop accumulation, increased DNA damage markers, and impaired repair, suggesting a potential causal role in genome stability. On the other hand, CNV alterations frequently observed in tumors may elevate gene expression through cis-dosage effects, indicating that high FMR1 expression in malignant cells may partly result from local CNV events or a broader aneuploid background [50, 51]. Therefore, rigorous causal validation experiments—such as FMR1 manipulation combined with γH2AX/53BP1 foci assays, comet assays, and R-loop (S9.6) analyses—represent important directions for future investigation. The analysis generated a detailed atlas of the gastric cancer tumor microenvironment (TME), identifying major cell types including epithelial cells, fibroblasts, and various immune cell populations. Notably, immune cells accounted for a substantial proportion of the TME (about 60%), predominantly comprising T cells and myeloid cells, which is consistent with the immunosuppressive characteristics of gastric cancer and provides a foundation for exploring potential immunotherapeutic targets.

Malignant epithelial cells were identified using inferCNV, which revealed FMR1 enrichment. Subsequent GSVA demonstrated consistent upregulation of stemness-, chemoresistance-, and immune evasion–related pathways in FMR1-high cells, suggesting an association with highly proliferative and invasive phenotypes, our in vitro experiments confirmed these findings.

At the molecular level, We performed RNA-seq and identified five downstream regulatory genes—c-MYC, MAD2L1, TFDP1, CDK2, and CDK4. Among them, MAD2L1 is a core component of the spindle assembly checkpoint and plays a critical role in ensuring accurate chromosome segregation and maintaining genome stability; its aberrant activation can cause chromosomal instability, promote uncontrolled proliferation, and drive tumor progression [52]. TFDP1 also contributes to tumor development, where its abnormal activation often leads to sustained transcription of E2F target genes, thereby promoting G1–S phase transition and unlimited proliferation [53]. Dysregulated activation of the cyclin-dependent kinases CDK2 and CDK4 has likewise been closely linked to tumorigenesis, with recent studies [54, 55] showing that persistent CDK2 and CDK4 activation not only supports tumor cell proliferation, invasion, and metastasis but may also contribute to chemoresistance, making them important therapeutic targets. Together, these findings suggest that FMR1 may influence tumor progression through multiple downstream effectors; however, we focused on the c-MYC pathway as the downstream regulatory axis of greatest interest for further investigation. The Myc protein is encoded by the proto-oncogene c-Myc, a member of the Myc family that also includes N-Myc and L-Myc [56]. c-Myc is an oncoprotein comprising 439 amino acids, featuring a C-terminal DNA-binding domain, a central region, and an N-terminal transcription activation domain (TAD) [57]. It is ubiquitously expressed at high levels in proliferating cells and is tightly regulated by a variety of upstream and downstream mechanisms at the genetic, protein, and mRNA levels [58]. As a critical transcription factor, c-MYC plays a pivotal role in the initiation and progression of numerous cancers [59, 60]. The c-MYC proto-oncogene is implicated in the pathogenesis of most human tumor types, reinforcing many of the classical “hallmarks” of cancer, including tumor growth driven by altered DNA replication and transcription, enhanced cell proliferation and growth, increased protein synthesis, and metabolic reprogramming [61]. Functionally, c-Myc acts primarily as a transcriptional regulator and has been described as a “master gene regulator,” exhibiting aberrant activation in a wide range of malignancies, including hematological cancers [62] as well as solid tumors such as breast [63], pancreatic [64], colorectal [65], and prostate cancers [66].

Our data indicate that FMR1 promotes the oncogenic properties of gastric cancer cells by directly activating c-MYC.. Additionally, prior research has shown that c-MYC induces the invasion and migration of gastric cancer cells by upregulating CDC25B and downregulating YWHAE expression [67]. c-MYC also promotes gastric cancer cell proliferation, migration, invasion, and epithelial–mesenchymal transition (EMT) in vitro, as well as tumor growth in vivo, by interacting with and activating downstream lncRNAs [59]. In our study, we demonstrated that FMRP directly interacts with c-MYC and regulates its expression by stabilizing the protein through suppression of proteasomal degradation, thereby enhancing its activity and promoting malignant phenotypes in gastric cancer cells.

Given our finding that FMR1 promotes malignant phenotypes of gastric cancer cells through activation of the c-MYC pathway, targeting key molecules along this axis may represent a potential therapeutic strategy. Recent studies have developed FMRP-specific proteolysis-targeting chimeras (PROTACs) capable of inducing FMRP degradation, which showed beneficial effects on the tumor immune microenvironment [68]. Other work has identified FMRP as a potential target for cancer immunotherapy, as it regulates immune cell metabolism, suggesting that inhibition of FMRP could overcome resistance to anti–PD-1, anti–PD-L1, and anti–CTLA-4 therapies. Small-molecule inhibitors of the RNA-binding domain of FMRP may enhance T-cell metabolism and sensitize tumors to immune checkpoint inhibitors (ICIs) [69]. Similarly, therapeutic strategies against c-MYC are rapidly advancing. For example, Omomyc has been shown to antagonize c-MYC–mediated transcriptional activity and induce sustained tumor regression across multiple tumor models, becoming the first MYC inhibitor to enter clinical trials in patients with advanced solid tumors—providing proof of concept that MYC inhibition is a viable anticancer strategy [70]. In addition, indirect approaches such as BET inhibitors, which downregulate c-MYC expression, have also demonstrated promise in Myc-driven tumors [71]. Nonetheless, several challenges remain for clinical translation. First, direct inhibition of FMR1 as an RNA-binding protein is still in the exploratory stage. Second, c-MYC has long been regarded as “undruggable,” with its structural features posing obstacles to small-molecule inhibitor development. Finally, gastric cancer is characterized by pronounced molecular heterogeneity and a complex immune microenvironment, which may affect the efficacy of targeting the FMR1/c-MYC axis. Future advances will likely require integration of molecular subtyping with rational combination therapies to fully exploit the clinical potential of this strategy.

A limitation of our study is that the mechanistic insights into FMR1’s role were primarily derived from bioinformatic analyses and in vitro experiments, lackingfurther investigation in animal models. In addition, for the clinical samples used in immunohistochemistry, a total of 78 patient specimens were selected according to the inclusion and exclusion criteria. However, because pathological records for some cases were incomplete, further subtype stratification could not be performed. As a result, Lauren classification was not applied in this study, and potential differences in FMR1 expression patterns between intestinal- and diffuse-type gastric cancers could not be evaluated. This limitation may have introduced confounding factors to some extent. Future studies are therefore needed to confirm the clinical significance of FMR1 as a prognostic biomarker in gastric cancer using additional patient cohorts and to explore its role within the gastric cancer immune microenvironment.

In conclusion, this study reveals the oncogenic role of FMR1 in gastric cancer and demonstrates that it promotes cell proliferation, migration, and invasion via the c-MYC pathway, providing new insights into FMR1 as a potential therapeutic target for gastric cancer.

## Supplementary Information

Additional file 1.

Additional file 2.

Additional file 3.

Additional file 4.

## Data Availability

During this study, some of the data generated or analyzed are included in this article and its supplementary information files. Other data sets are available from the corresponding author upon reasonable request.
