# Supplementary figures and images for: Fragile X mental retardation 1 gene FMR1 promotes proliferation, migration, and invasion of gastric cancer cells via c-MYC
Source: J Transl Med. 2025 Nov 3;23:1210. doi: 10.1186/s12967-025-07140-8 (PMC12581516; doi:10.1186/s12967-025-07140-8)

A

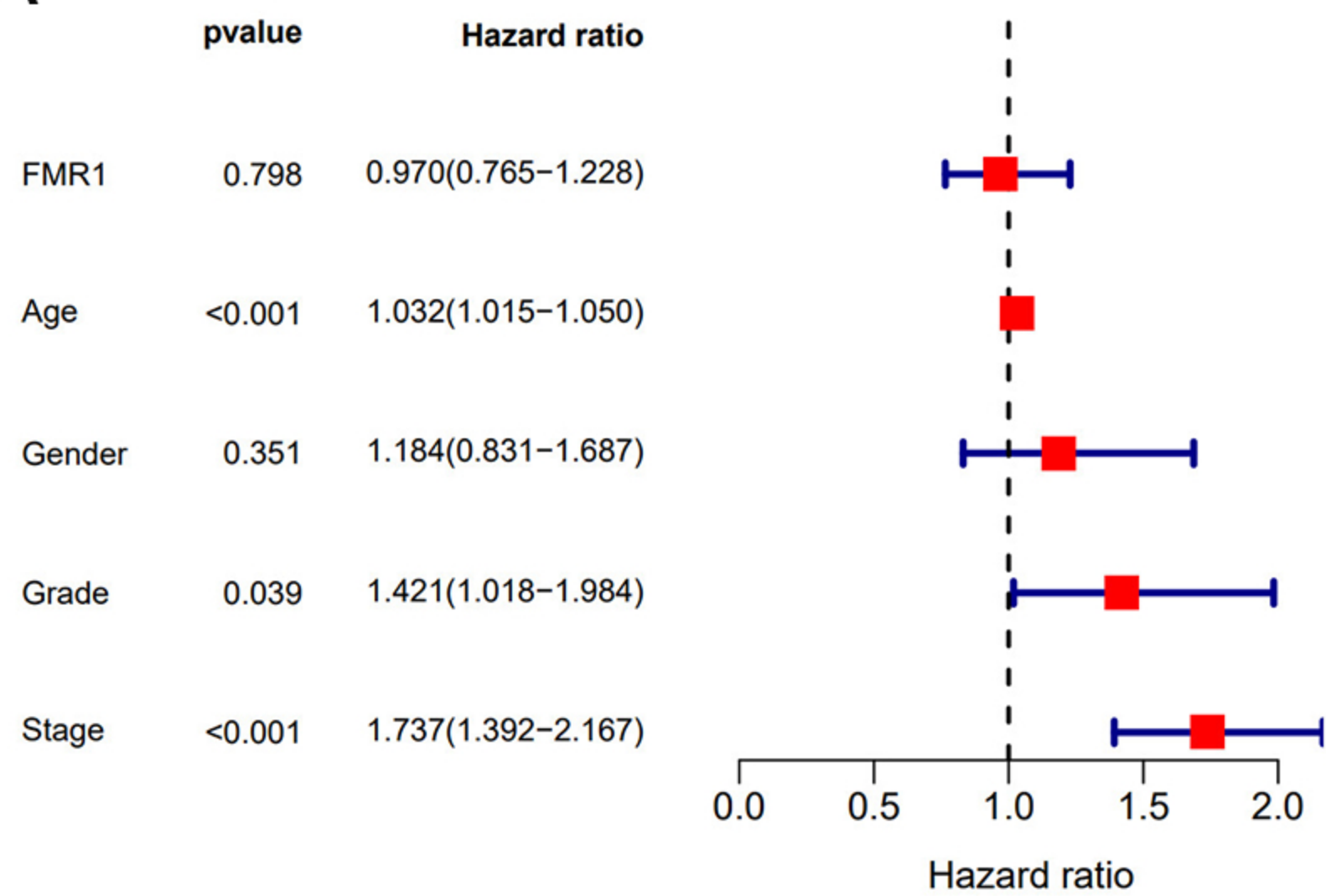

B

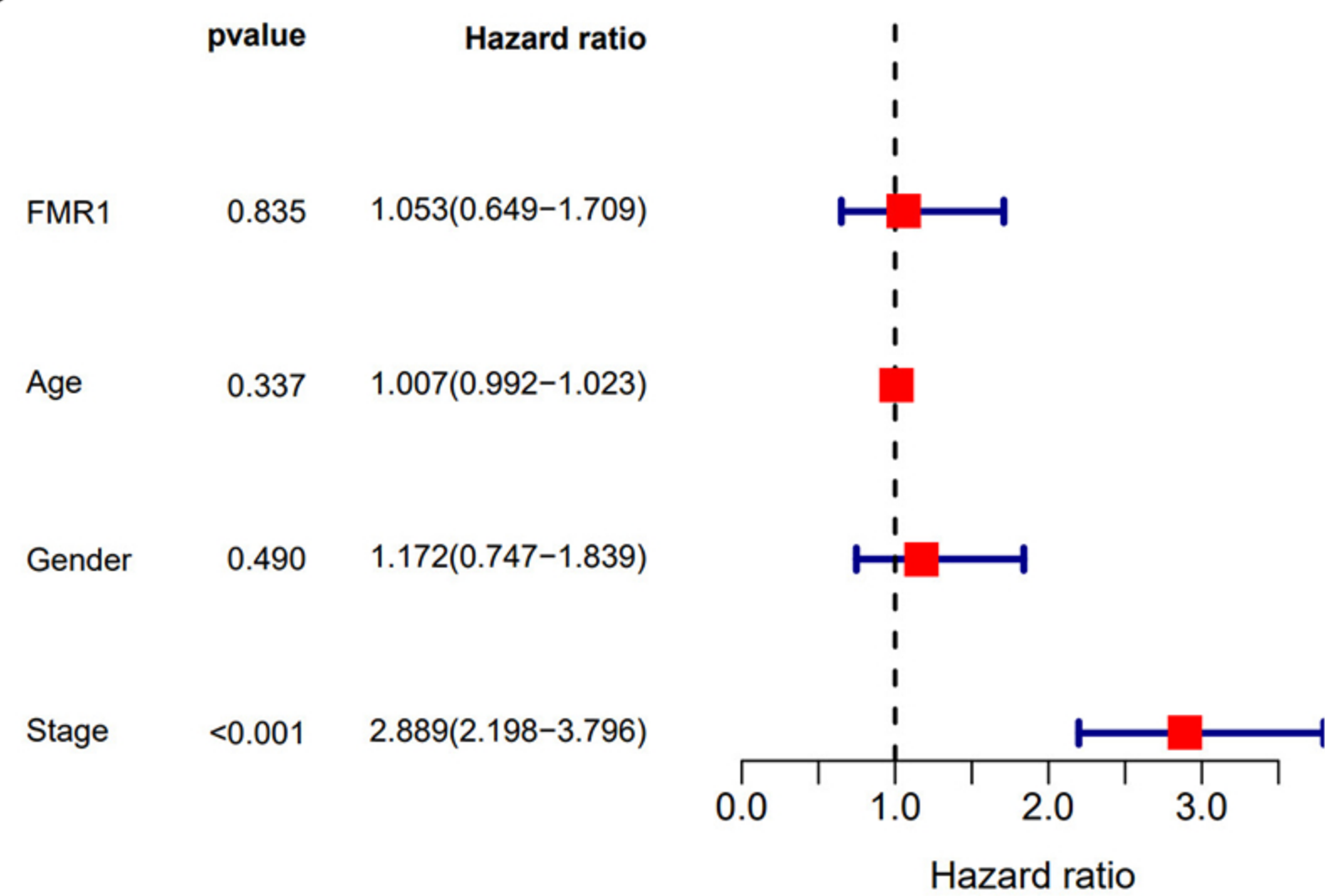

C

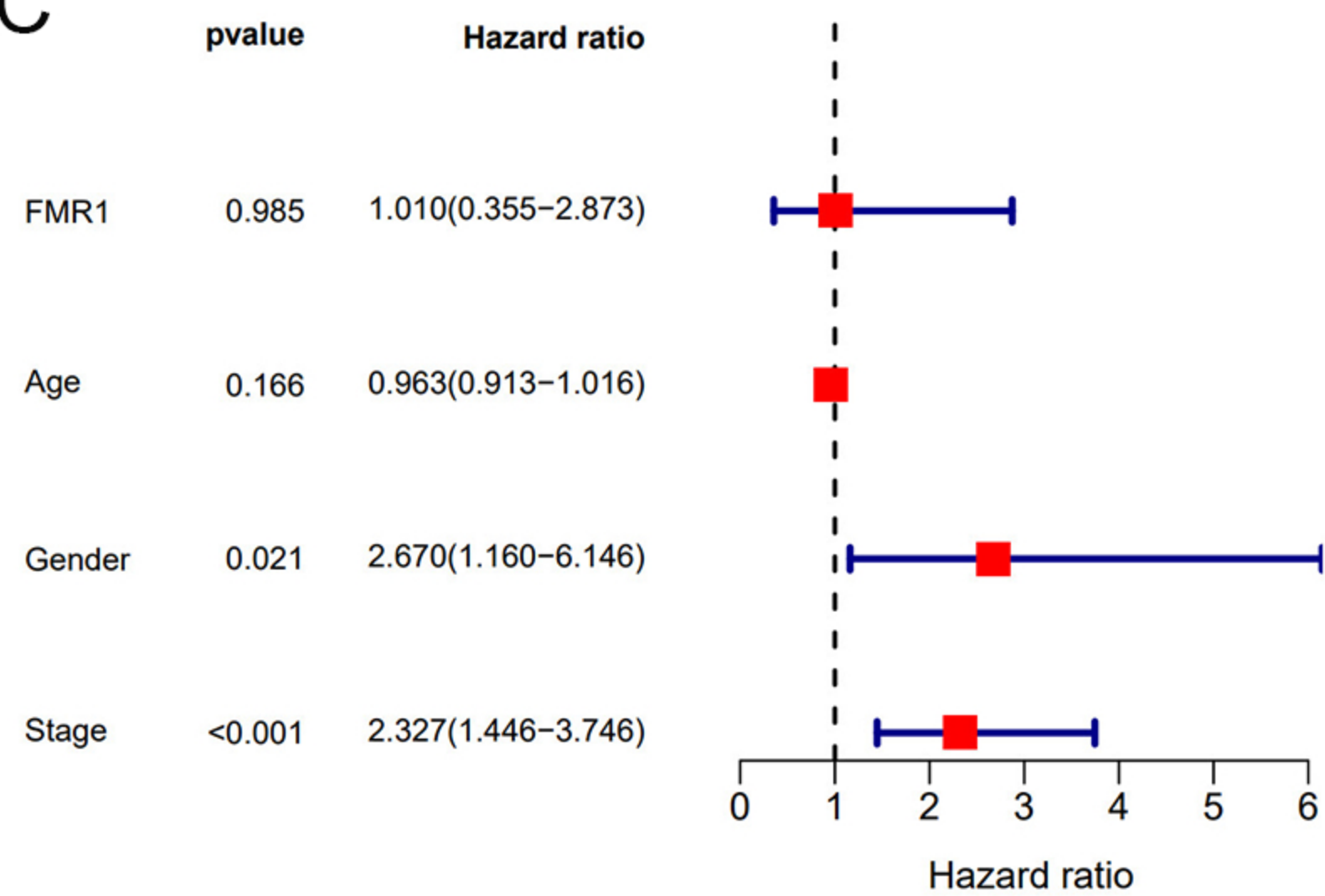

D

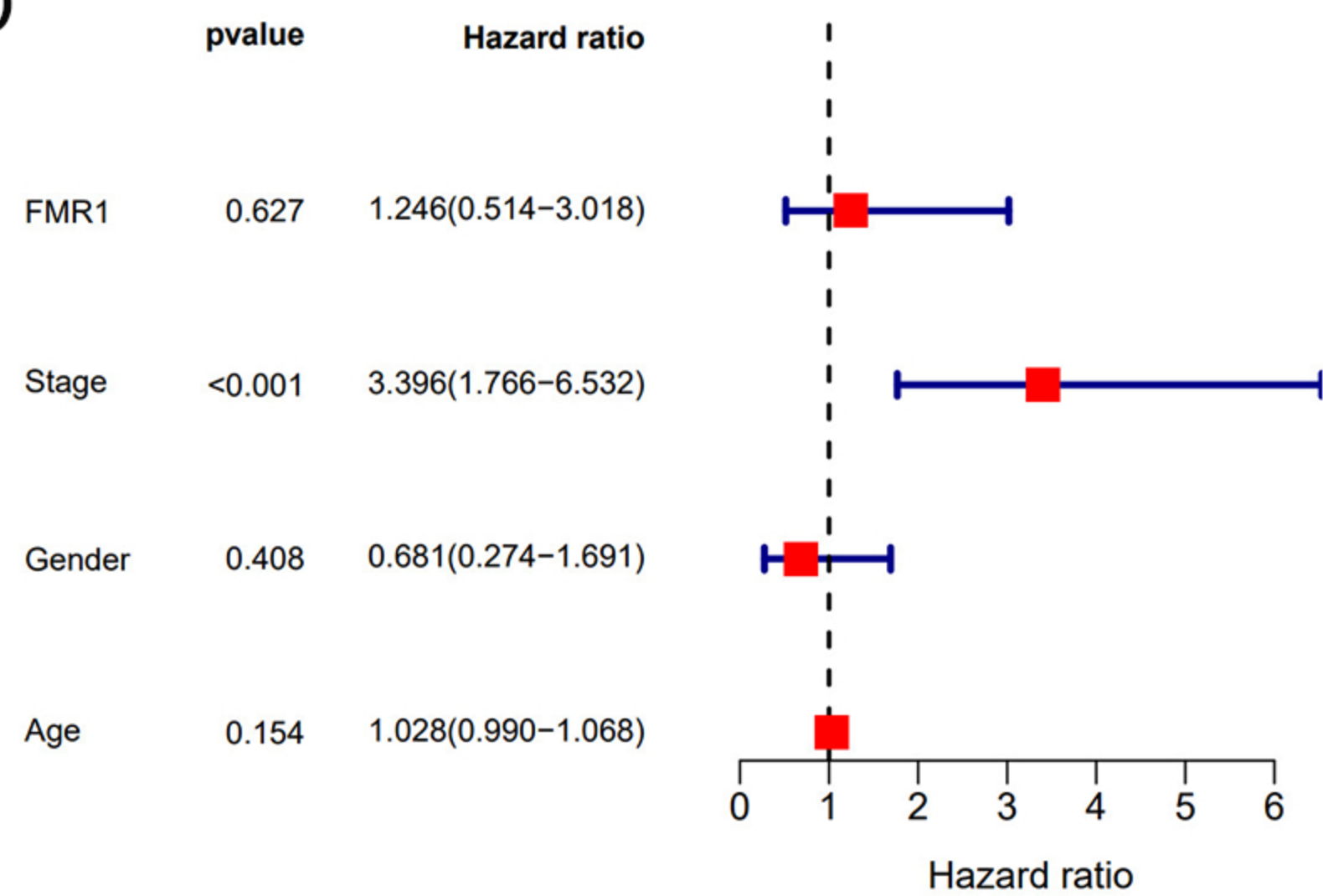

E

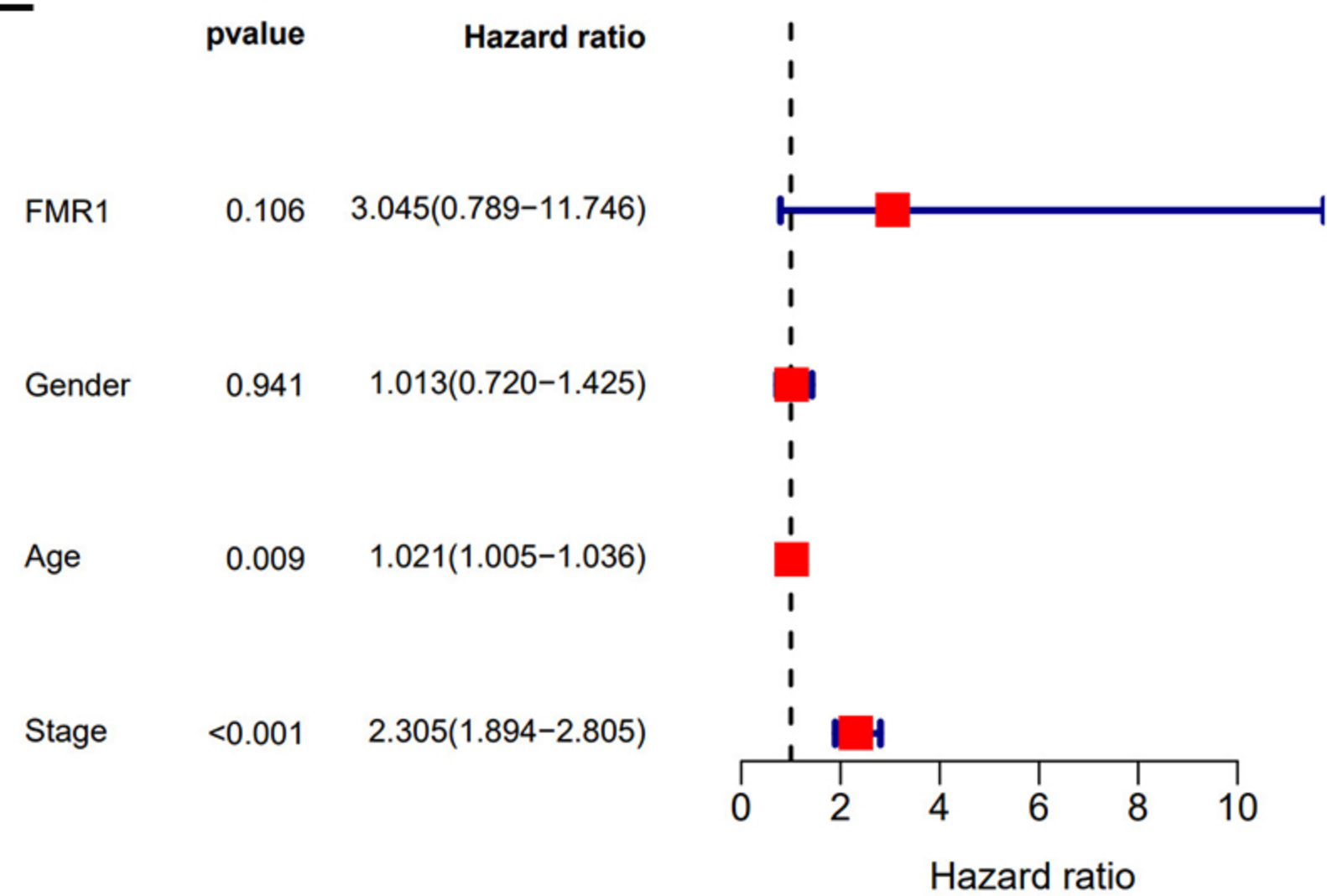

F

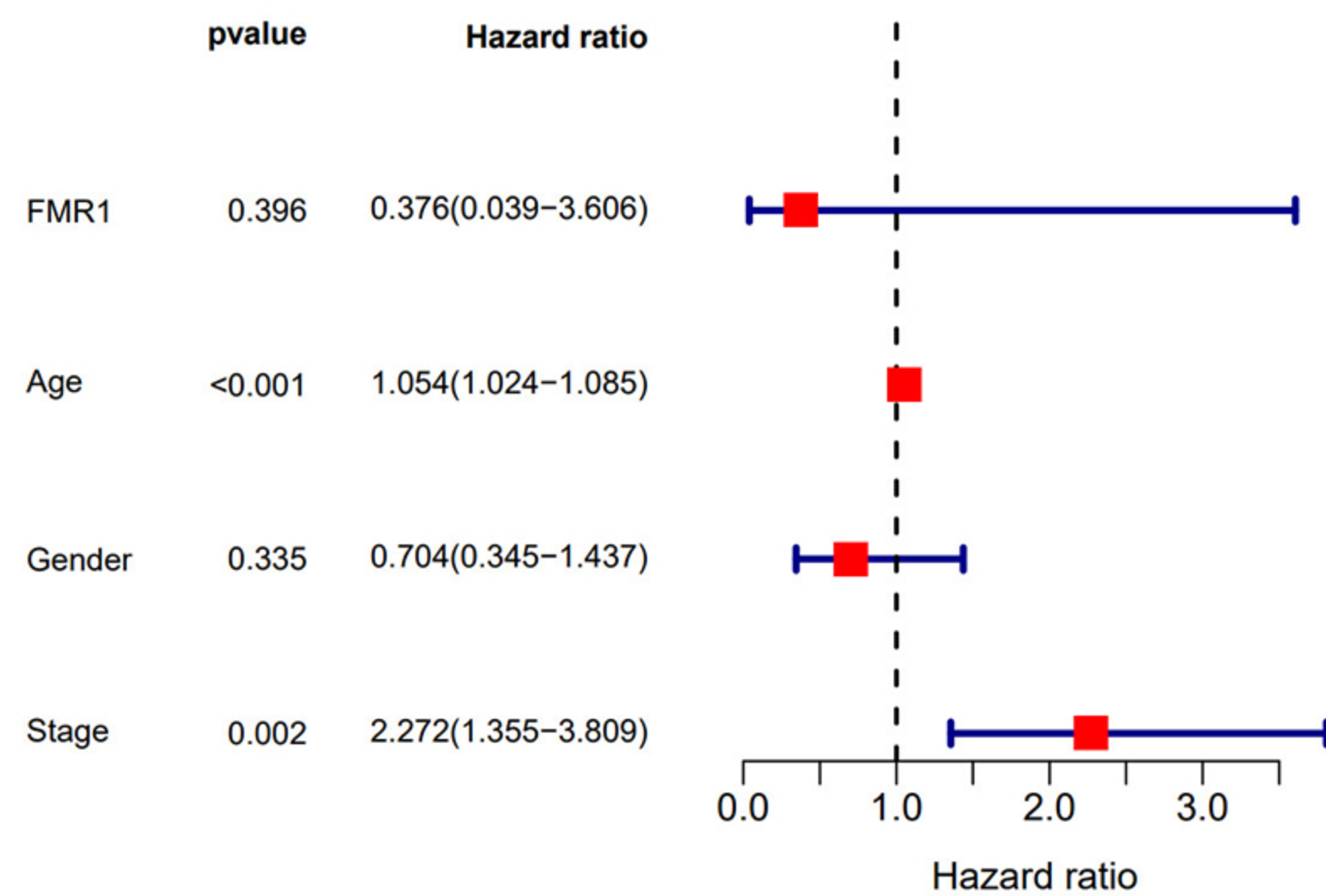

Supplement: Supplementary file 1 — Additional file 1. [file 12967_2025_7140_MOESM1_ESM.pdf]

A

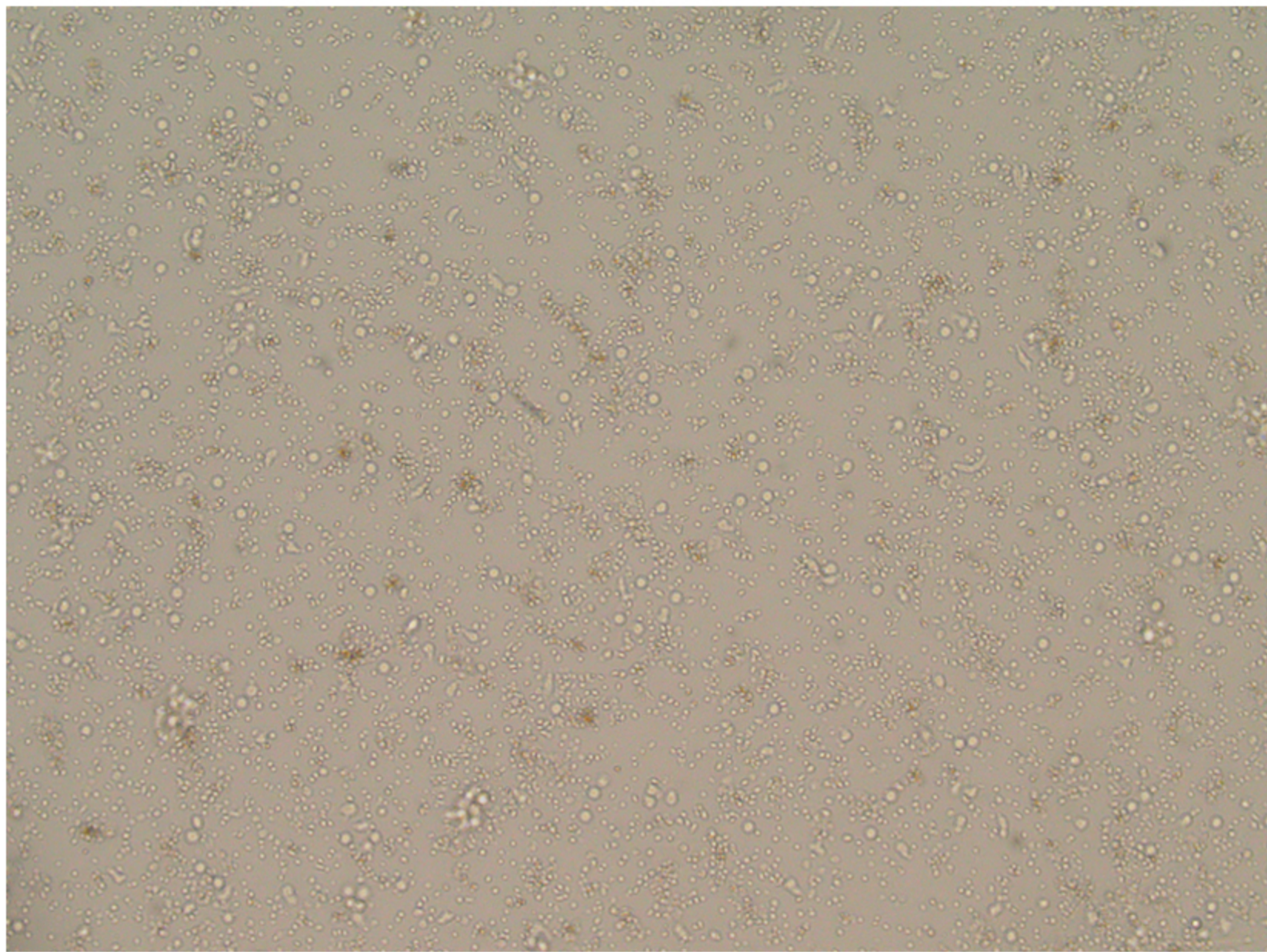

B

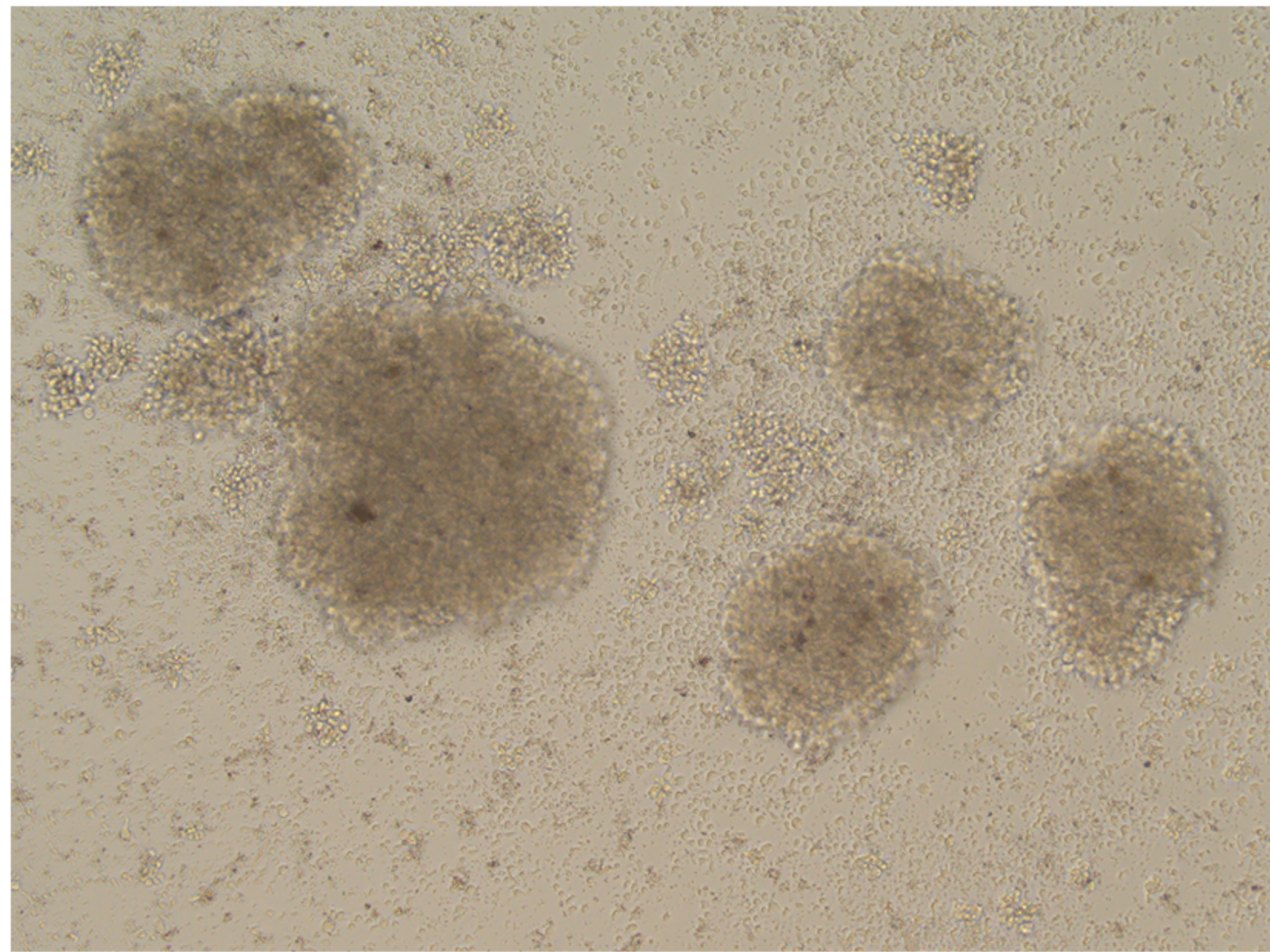

Supplement: Supplementary file 2 — Additional file 2. [file 12967_2025_7140_MOESM2_ESM.pdf]
